# Interaction of CYP3A4 with Rationally Designed Ritonavir Analogues: Impact of Steric Constraints Imposed on the Heme-Ligating Group and the End-Pyridine Attachment

**DOI:** 10.3390/ijms23137291

**Published:** 2022-06-30

**Authors:** Eric R. Samuels, Irina F. Sevrioukova

**Affiliations:** 1 Department of Pharmaceutical Sciences, University of California, Irvine, CA 92697, USA; samuelse@uci.edu; 2 Department of Molecular Biology and Biochemistry, University of California, Irvine, CA 92697, USA

**Keywords:** CYP3A4, inhibitor design, ritonavir analogues, crystal structure, structure–activity relationship

## Abstract

Controlled inhibition of drug-metabolizing cytochrome P450 3A4 (CYP3A4) is utilized to boost bioavailability of anti-viral and immunosuppressant pharmaceuticals. We investigate structure–activity relationships (SARs) in analogues of ritonavir, a potent CYP3A4 inhibitor marketed as pharmacoenhancer, to determine structural elements required for potent inhibition and whether the inhibitory potency can be further improved via a rational structure-based design. This study investigated eight (series VI) inhibitors differing in head- and end-moieties and their respective linkers. SAR analysis revealed the multifactorial regulation of inhibitory strength, with steric constraints imposed on the tethered heme-ligating moiety being a key factor. Minimization of these constraints by changing the linkers’ length/flexibility and N-heteroatom position strengthened heme coordination and markedly improved binding and/or inhibitory strength. Impact of the end-pyridine attachment was not uniform due to influence of other determinants controlling the ligand-binding mode. This interplay between pharmacophoric determinants and the end-group enlargement can be used for further inhibitor optimization.

## 1. Introduction

Humans have multiple cytochrome P450 (CYP) enzymes involved in the oxidation of endogenous molecules and xenobiotics. Drug-metabolizing CYP3A4 is the most abundant hepatic and intestinal CYP isoform that clears over half of administered pharmaceuticals through oxidation [1,2]. The 57 kDa protein has a large and malleable active site that can accommodate structurally diverse compounds, sometimes two or more at the same time [3]. Drugs can also activate and inhibit CYP3A4 [4], which is subject to both reversible and irreversible (mechanism-based) inhibition.

**Figure 1:**
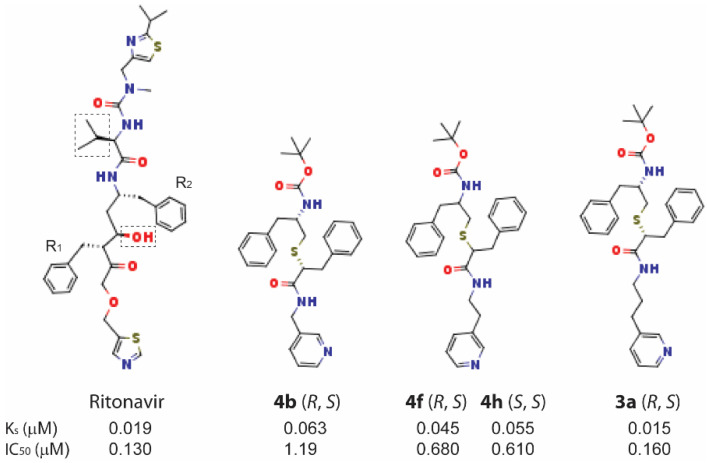
Chemical structures of ritonavir and previously reported analogues from series III (**4b**, **4f**, and **4h**) [16] and V (**3a**) [17] that were used for structural and functional comparison in this study. The R_1_- and R_2_-phenyl side-groups are indicated. If the central hydroxyl group of ritonavir is removed, the phenyl side-groups would be in the *R*/*R* configuration. The listed IC_50_ values were measured for the BFC debenzylation activity of CYP3A4 using the same method as in this study. In cobicistat, a close derivative of ritonavir, there are only two modifications (boxed): the backbone hydroxyl group is removed, and the valine chain in the end-moiety is replaced with the bulkier morpholine ring.

CYP3A4 inhibition is usually undesired because it could lead to drug toxicity, drug–drug interactions, and other serious adverse effects. In some cases, however, a partial loss of CYP3A4 activity can be beneficial, as it could slow down metabolism of co-administered drugs and increase their blood levels. This pharmacoenhancing (boosting) effect is currently utilized in anti-HIV and anti-HCV therapy, where a potent CYP3A4 inhibitor, ritonavir (Figure 1), or its derivative cobicistat is co-administered with antiviral drugs to prevent their quick metabolism and improve therapeutic efficiency [5,6,7]. Another potent CYP3A4 inhibitor, the antifungal agent ketoconazole, is co-prescribed with immunosuppressants in organ transplant recipients to reduce drug dosage and lower the cost of post-transplantation therapy [8,9]. Until recently, ketoconazole was also widely used as an index inhibitor of CYP3A4 for in vitro and in vivo drug–drug interaction studies but, due to its high hepatotoxicity, its usage is now limited [10]. Ritonavir has several disadvantages as well, including its multiple off-target activities and poor physicochemical properties [11]. Cobicistat lacks anti-viral activity and has higher bioavailability and better tolerability [12], but inhibits CYP3A4 less potently than ritonavir and cross-reacts with other drug-metabolizing CYPs [13]. Importantly, none of the marketed pharmacoenhancers were rationally designed or developed based on the CYP3A4 crystal structure. The CYP3A4 inhibitory activity of ritonavir and ketoconazole was coincidental [5,14], whereas cobicistat was developed based solely on chemical structure–activity relationship (SAR) analysis [15].

Ritonavir remains one of the most potent CYP3A4 inhibitors in clinical use. Therefore, we undertook a series of studies on its analogues to better understand the mechanistic basis for the inhibitory action. Ritonavir is generally thought to act as a mechanism-based inhibitor by producing reactive metabolites that inactivate CYP3A4 via covalent attachment [18,19,20,21,22]. However, in our soluble reconstituted system, we could not detect time-dependent inactivation of CYP3A4 after its pre-incubation with cytochrome P450 reductase, NADPH, and ritonavir and, instead, observed an increase in IC_50_, likely due to partial metabolism of ritonavir [16]. It remains to be established whether the membrane bilayer, cytochrome b_5_, and/or other microsomal proteins are required for reactive metabolite production. Regardless, our goal is to identify structural elements critical for strong binding and potent inhibition of CYP3A4 by studying SARs in analogues lacking the isopropyl thiazole group, a possible site of ritonavir’s reactive metabolite formation [18,22]. This knowledge could guide the development of more potent pharmacoenhancers, as well as help medicinal chemists to design safer, more effective therapeutic agents by eliminating the undesired CYP3A4 inhibitory potential in drug candidates early in the discovery process. Our work is also important from the basic science perspective, as it would lead to a better understanding of the conformational flexibility of CYP3A4 and its adaptive responses for ligand binding.

The first sets of inhibitors that we investigated contained ritonavir, desoxyritonavir, and their closest analogues [23,24,25,26]. Based on the spectral and inhibitory properties and the ligand-binding modes, we concluded that coordination to the heme is the driving force for the association of ritonavir-like molecules, with the pyridine moiety identified as the strongest heme-ligating group. These initial studies allowed us to build the first structure-based pharmacophore model for a potent CYP3A4 inhibitor [27]. To test and optimize the pharmacophore, we utilized a build-from-scratch approach by gradually attaching various backbones and side-groups to the pyridine ring. Five series of inhibitory compounds have been designed thus far to study the impact of the backbone length/composition, the size/hydrophobicity/stereochemistry of the R_1_/R_2_ side-groups (represented by phenyls in ritonavir; Figure 1), and the length of the pyridyl–R_1_ and R_1_–R_2_ spacers [16,17,28,29,30]. There was no strong correlation between the binding affinity and inhibitory potency of the investigated compounds, but both parameters were improved with an increase in side-group hydrophobicity and the length of the pyridyl–R_1_ spacer (up to seven-atom separation). Side-group stereochemistry was less impactful, though CYP3A4 was found to favor compounds in the R/S, but not S/R, configuration.

**Figure 2:**
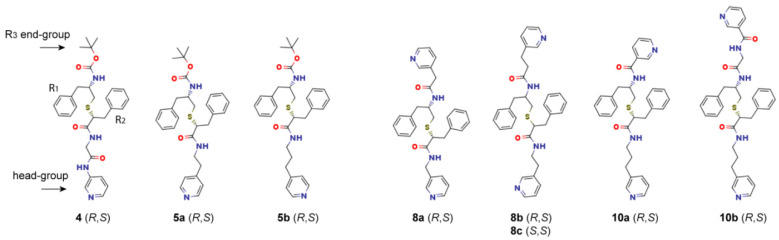
Chemical structures of series VI analogues. The pyridine head-group, R_1_/R_2_-phenyl side groups and their stereo configuration, and terminal R_3_ moiety are indicated.

This study was designed to elucidate if/how the binding and inhibitory strength is affected by the heme-ligating N-pyridine position, flexibility of the head-pyridine spacer, and attachment of the end-pyridine (R_3_-moiety). The new set contained eight inhibitors (series VI; Figure 2), all of which had R_1_/R_2_-phenyls, to enable comparisons with the previously reported series III and V counterparts, **3a**, **4b**, **4f**, and **4h** (Figure 1) [16,17]. Spectral, functional, and structural data on the series VI compounds confirmed the multifactorial regulation of the inhibitory strength, with steric constraints imposed on the tethered head-group found to be the key factor. Minimization of these constraints was strictly required for the potent inhibition of CYP3A4 and could be achieved through an increase in the length/flexibility of the head-group linker, *meta*-to-*para* switch in the N-pyridine position, and changes in the R_1_/R_2_ configuration. Attachment of the end-pyridine, in turn, had no uniform effect and led to an increase or decrease in K_s_, IC_50_, or both, depending on the head/end-group spacers and stereochemistry. Together, our data demonstrate the importance of the interplay between pharmacophoric determinants in controlling the binding/inhibitory strength and ligand binding orientation.

## 2. Results

### 2.1. Rationale for Series VI Analogues

Our previous study showed that one-atom extension of the pyridyl–ethyl spacer markedly improved the binding affinity and inhibitory potency of ritonavir-like compounds [17]. The head-group linker elongation allowed inhibitors to adopt a more relaxed conformation and led to stronger heme ligation, which was evident from the shorter and near-perpendicular Fe–N bond. To gain further insight into how the pyridine–R_1_ spacer affects the inhibitory potency and ligand-binding mode, we synthesized **4** as a counterpart of **3a**, the most potent inhibitor from the previous series (Figure 1) [17], by replacing the flexible aliphatic spacer with a rigid peptide bond (Figure 2). Compounds **5a** and **5b**, in turn, were designed with a traditional pyridyl–ethyl or pyridyl–propyl spacer, respectively, but the heme-ligating N-heteroatom was moved from the *meta*- to *para*-position. It was anticipated that comparison of **5a**–**b** with the *meta*-N-containing **4f** and **3a** (Figure 1) would clarify if/how positional changes in the electron-donating nitrogen atom affect the inhibitory potency and binding mode. Finally, as a first step toward optimization of the terminal group, we synthesized five compounds where the protective *tert*-butyloxycarbonyl (Boc) group, which served as the R_3_ end moiety, was substituted with a *meta*-N-pyridine linked through various spacers. The first three bi-pyridine compounds were near-symmetrical and contained either a pyridyl–methyl (**8a**) or pyridyl–ethyl linker (**8b** and **8c**). Although the *R*/*S* side-group configuration was found to be the most favorable in binding CYP3A4, a few *S*/*S* stereoisomers were among the most potent inhibitors as well [17,30]. Therefore, we included **8c** (S, S) along with **8b** (*R*, *S)* to assess the impact of stereochemistry. The last two compounds, **10a** and **10b**, had the most optimal pyridyl–propyl head-group spacer, while the end-pyridine was linked via a single or double amide bond, respectively (Figure 2). This allowed us to test the impact of the length of the R_2_–R_3_ spacer.

### 2.2. Interaction of CYP3A4 with Compound ***4***

#### 2.2.1. Spectral and Inhibitory Properties of **4**

**Figure 3:**
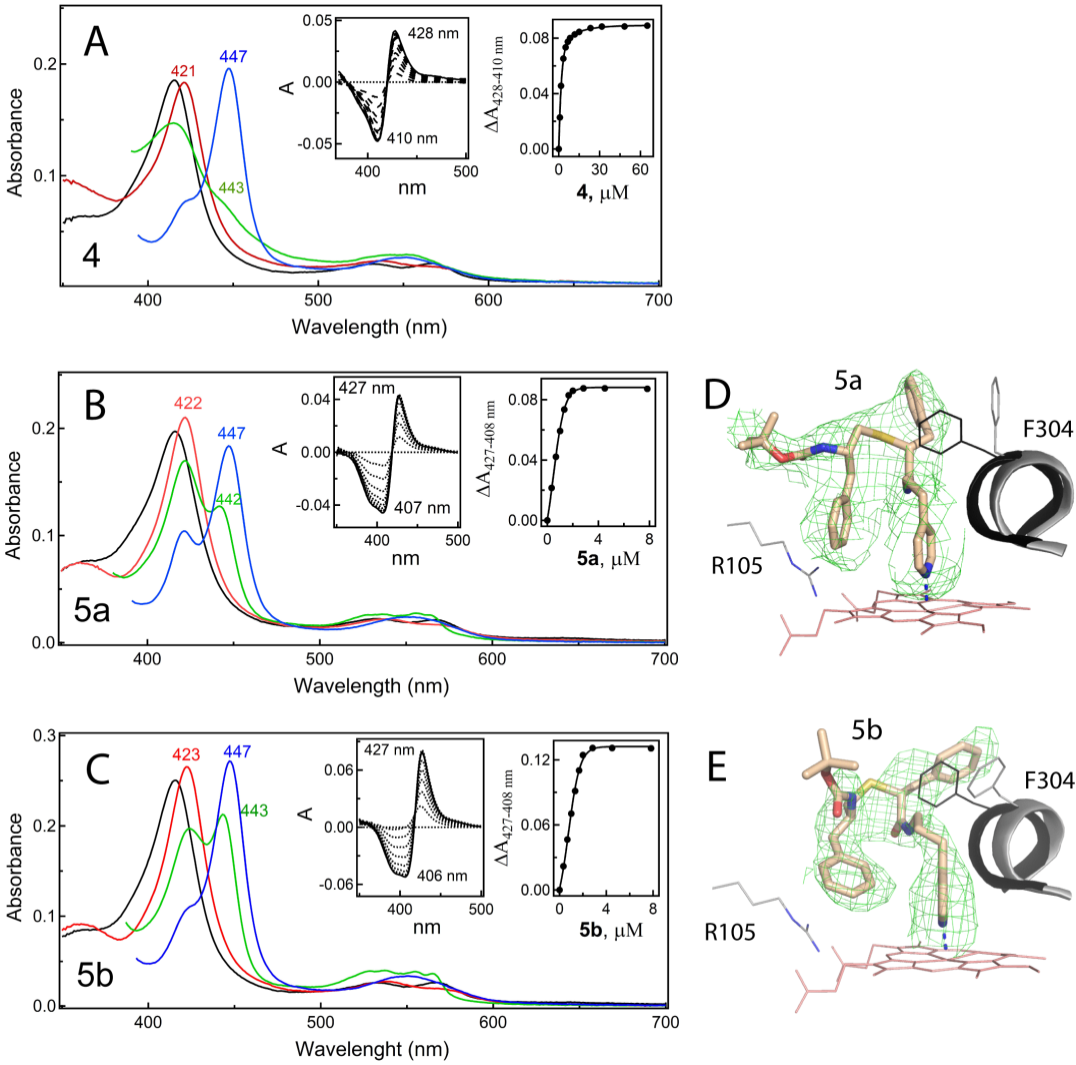
Spectral properties of **4** and **5a,b** and the binding modes of the latter two compounds. (**A**–**C**) Ligand-induced spectral changes in CYP3A4. Absorbance spectra of ferric ligand-free and inhibitor-bound CYP3A4 are in black and red, respectively. Spectra of ferrous ligand-bound CYP3A4 and its CO adducts are in green and blue, respectively. Left insets are the difference spectra recorded during equilibrium titrations; right insets are the titration plots with quadratic fittings. The derived spectral dissociation constants (K_s_) are given in Table 1. (**D**,**E**) Crystallographic binding modes of **5a** and **5b**, respectively. Part of the adjacent I-helix and Phe304 in the inhibitory complexes and water-bound CYP3A4 (5VCC structure) are depicted in gray and black, respectively. Polder omit electron density maps contoured at 3σ level are shown as green mesh. The **5a**- and **5b**-bound structures of CYP3A4 have the PDB ID codes 7UF9 and 7UFA, respectively.

As expected, compound **4** induced a red shift in the Soret band, indicative of the N-pyridine ligation to the heme iron (type II spectral change; Figure 3A). However, compared to its closest analogue, **3a** (Figure 1) [17], λ_max_ for the ferric **4**-bound CYP3A4 was at a shorter wavelength (421 nm vs. 422 nm), and the A_421/417_ ratio and ΔA_max_ (maximal peak/trough amplitude in the difference spectra) were considerably lower (Table 1). Moreover, the ferrous **4**-bound CYP3A4 did not produce a 442 nm absorption peak, characteristics of ritonavir-like compounds. These spectral features suggest a decrease in the electronic orbital overlap, leading to a weaker σ-donor nitrogen ligation, possibly due to geometric constraints imposed by the rigid spacer.

Spectral dissociation constants (K_s_; measure of the binding affinity) for **4** and other inhibitors were calculated from the titration plots (left insets in Figure 3A–C). Strikingly, relative to **3a**, the linker rigidification in **4** led to a 100-fold increase in K_s_ (from 0.015 μM to 1.36 μM) and 10-fold increase in IC_50_ for the 7-benzyloxy-4-(trifluoromethyl) coumarin (BFC) *O*-debenzylase activity of CYP3A4 (from 0.16 μM to 1.6 μM). Another marked difference was in thermostability of the ligand-bound CYP3A4, assessed by measuring changes in the melting temperature (T_m_). The binding of **4** only modestly enhanced protein stability, with a T_m_ increase of 1.9 °C vs. 8.1 °C for **3a**, one of the best stabilizers of CYP3A4.

#### 2.2.2. Ligand-Binding and Heme-Bleaching Kinetics

The kinetics of ligand binding were measured by monitoring the conversion of the heme iron to the low-spin form. As observed for other analogues, the association of CYP3A4 with **4** was biphasic within the studied time interval (Figure S1). Moreover, the reaction was virtually unaffected by the increased rigidity of the head-group linker, as the rate constant and the fraction of the fast phase (13.8 s^−1^ and 27%, respectively) were well within the range derived for analogues from this and the previous series (Table 2). Thus, compound **4** can access and ligate to the heme as fast as **3a**.

Ritonavir-like molecules inhibit CYP3A4 not only via strong heme ligation, but also through the spatial filling of the active site, which prevents substrates from accessing the catalytic center. Previously, we showed that the heme accessibility in the inhibitor-bound CYP3A4 can be probed using a small oxidizing agent, hydrogen peroxide [17]. The H_2_O_2_-dependent heme bleaching assay was utilized for series-VI compounds and revealed that **4** had the smallest heme-protective effect, with nearly half of the heme destroyed within the studied time interval (Table 2). That the CYP3A4–**4** complex could not be crystallized due to its dissociation under all tested conditions was another indication that **4** was a very weak binder**.** Together, these findings allow us to conclude that an increase in the rigidity of the pyridyl–linker does not eliminate the heme-ligating ability, but imposes steric constraints that disallow the pyridine moiety to optimally orient and maximize electronic orbital overlap with the heme cofactor. This weakens the Fe–N bond and, subsequently, the inhibitory complex.

### 2.3. Interaction of CYP3A4 with ***5a*** and ***5b***

#### 2.3.1. Spectral, Kinetic, and Inhibitory Properties of **5a,b**

The *meta*-to-*para* switch in the pyridine N-heteroatom did not diminish the heme-binding ability of compounds **5a**,**b**. On the contrary, compared with the *meta*-N-containing **4f** and **3a** (Figure 1), the λ_max_ and ΔA_max_ values derived for the **5a**/**b**-bound CYP3A4 (Table 1) were larger and indicative of more optimal/stronger heme coordination [31]. Furthermore, **5a**,**b** were the fastest binders in the series and could effectively protect the heme from H_2_O_2_ bleaching (22% heme destroyed vs. 37% for **4f** and 18% for **3a**; Table 2). However, compared to the *meta*-N-containing counterparts, an improvement in the binding affinity and inhibitory potency was observed only for **5a** (2.2- and 1.3-fold increase, respectively, relative to those for **4f**). Likewise, **5a**-bound CYP3A4 had greater thermostability than the **4f**-bound form (ΔT_m_ of 5.2 °C and 4.2 °C, respectively). For further comparison, ΔT_m_ for **5b**–CYP3A4 was 5.0 °C vs. 8.1 °C for **3a**. Thus, the beneficial effect of the *meta*-to-*para* N-pyridine switch varied depending on the length of the head-group linker. Structural data on **5a**/**b**–CYP3A4 complexes provided some insights into the interplay between the heme-ligating moiety and the connecting fragment.

#### 2.3.2. Crystal Structures of **5a**- and **5b**-Bound CYP3A4

**Figure 4:**
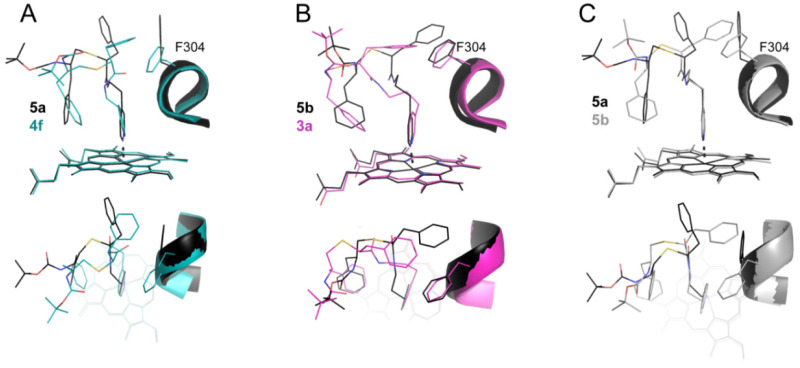
Structural comparison of **5a**–**4f** (**A**), **5b**–**3a** (**B**), and **5a**–**5b** pairs (**C**). The side and top views (upper and lower panels, respectively) illustrate differences in the backbone and side-group positioning. The central part of the I-helix is displayed to show the adaptive rotameric changes in Phe304. The **3a**- and **4f**-bound structures of CYP3A4 used for comparison have PDB ID codes of 7KVH and 6DAC, respectively.

**5a** and **5b** willingly co-crystallized with CYP3A4, and the resulting structures were solved to 2.45–2.50 Å resolution (Table S1). These and other inhibitors described below were fit into polder omit electron density maps (shown as the green mesh in the structural figures), the validity of which was verified (Table S3) [32]. Structural features of the inhibitory complexes are summarized in Table 3. The binding modes of **5a**,**b** relative to the central I-helix are shown in Figure 3D,E. Both compounds bind in a traditional orientation, with the R_1_-phenyl inserted into a hydrophobic pocket adjacent to the I-helix (P1 site) and the R_2_-phenyl above the heme, stacked between the heme-ligating pyridine and the propionate-binding Arg105 (P2 site). To better understand how changes in the pyridine nitrogen position affect the ligand-binding mode, a pairwise structural comparison was conducted (Figure 4).

Superposition of **5a**- and **4f**-bound CYP3A4 (Figure 4A) shows that the *meta*- to *para*-N switch largely affected the ligand-binding mode. The **5a** pyridine ring oriented near-perpendicular to the heme plane (5° vs. 20° incline angle for **4f**), and the Fe–N distance became considerably shorter (2.06 Å vs. 2.29 Å for **4f**). In accordance with the spectral data, this indicates more optimal/stronger heme coordination. Both inhibitors displaced the I-helix to the same degree (Table 3) but, due to distinct backbone curvatures, Phe304 and the side-groups adopted different conformations. As a result, only **5a** could establish an H-bond with Ser119. Moreover, the R_1_-phenyl in **5a** pointed toward, and protruded deeper into the P1 pocket, partially overlapping with Phe304, but in **4f**, it was oriented oppositely to Phe304 and without aromatic overlap. The **5a** R_2_-phenyl, in turn, was near-parallel to the heme-ligating pyridine, but had a 70° incline in **4f**. Nonetheless, both R_2_-groups were equally remote from the heme and Arg105, and only partially overlapped with the pyridine ring (Figure 3D,E). Because of the different incline angle, the **5a** R_2_-phenyl was positioned more favorably for cation–π interactions with Arg105. The terminal Boc-group was well-defined in **5a** and formed multiple Van der Waals interactions (Table 3) but disordered in **4f**. Collectively, these differences in heme coordination, H-bonding ability to Ser119, and interactions mediated by the side- and end-groups could explain why **5a** bound tighter and inhibited CYP3A4 more potently than **4f**.

For the **5b**–**3a** pair, the differences in the binding mode were less dramatic (Figure 4B): the pyridine rings nearly coincided, the Fe–N distance was comparable (2.08–2.11 Å), and the R_2_ and Boc-groups were at similar positions. Both inhibitors formed a strong H-bond to Ser119 and, despite the larger I-helix distortion by **3a** (>2 Å), Phe304 had the same rotameric conformation. One distinct feature of **5b** was the deeper embedment of its R_1_-phenyl into the P1 pocket, which enabled more extensive aromatic interactions with the nearby Phe213, Phe215, Phe304, and Phe241. Otherwise, there was a general similarity between the **5b** and **3a** binding modes, which could explain their equally strong binding affinity and inhibitory potency. The higher thermostability of **3a**-bound CYP3A4 might have resulted from the distinct incline and rotation angles of R_2_-phenyl, leading to the larger overlap with the heme and pyridine rings. Alternatively, the lower thermostability of the **5b**–CYP3A4 complex could be due to the larger backbone curvature and conformational strain caused by *para*-N coordination.

The comparison of the **5a**–**5b** pair (Figure 4C) helped to pinpoint structural differences caused by the head-group linker extension. The pyridine position and the Fe–N distance remained the same (Table 3), but marked differences in the Phe304 and R_1_ orientations were noticeable. In the elongated **5b**, the R_1_-phenyl adopted a different rotamer and deeply protruded into the P1 pocket, forcing Phe304 to swing away. The **5b** R_2_-phenyl, in turn, was perpendicular, rather than parallel, to the heme-ligating pyridine and was 1.0 Å closer, strengthening aromatic interactions. Together, these conformational distinctions could improve both the binding and inhibitory strength of **5b**.

### 2.4. Interaction of CYP3A4 with ***8a***, ***8b***, and ***8c***

#### 2.4.1. Spectral, Kinetic, and Inhibitory Properties of **8a–c**

The end-moiety of ritonavir, isopropyl-thiazole (Figure 1), serves as the primary site of CYP3A4-dependent metabolism [18]. This implies that ritonavir can enter the active site either with the head- or end-moiety first. This dual modality was suggested to underlie the biphasicity of the ligand-binding kinetics observed during association of CYP3A4 with ritonavir and its analogues [23,24,29]. Therefore, we hypothesized that the substitution of R_3_-Boc with a heme-ligating moiety, such as pyridine, might be advantageous, as it could lead to a productive heme coordination, regardless of the association mode. To test this possibility, we designed three compounds where *meta*-N-pyridine served as both the head- and end-group linked via one- (**8a**) or two-atom spacers (**8b** and **8c**; Figure 2). These near-symmetrical compounds were compared with the respective Boc-containing counterparts **4b**, **4f,** and **4h** (Figure 1) [16].

**Figure 5:**
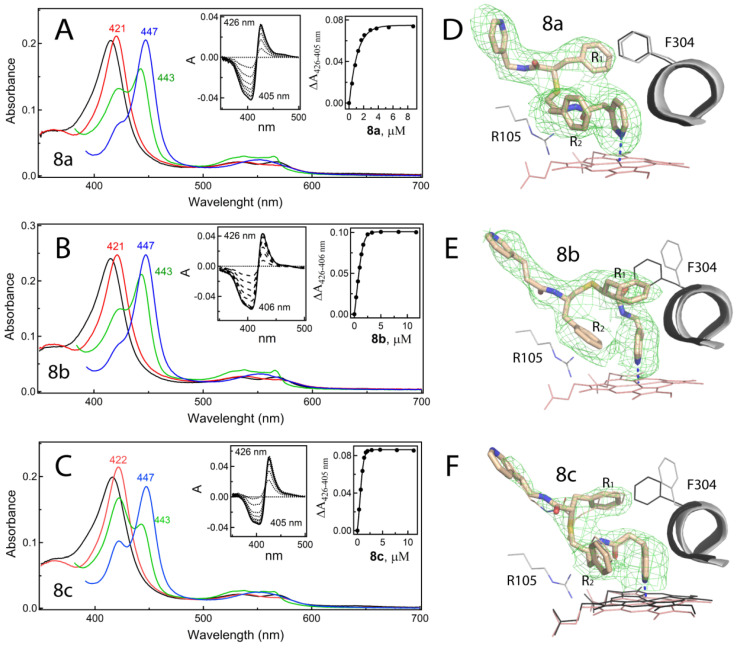
Spectral properties and the binding modes of **8a**–**c**. (**A**–**C**) Ligand-induced spectral changes in CYP3A4. Absorbance spectra of ferric ligand-free and inhibitor-bound CYP3A4 are in black and red, respectively. Spectra of ferrous ligand-bound CYP3A4 and its CO-adduct are in green and blue, respectively. Left insets are difference spectra recorded during equilibrium titrations; right insets are titration plots with quadratic fittings. The derived K_s_ values are given in Table 1. (**D**–**F**) Binding modes of **8a**–**c** observed in the crystal structures. The central portion of the I-helix and Phe304 in the inhibitory complexes and water-bound CYP3A4 (5VCC structure) are depicted in gray and black, respectively. **8a** and **8c** bind in a reverse mode, with the end-pyridine serving as the heme-ligating moiety. However, the R_1_- and R_2_-phenyl groups (indicated) occupy the same sites. Polder omit electron density maps contoured at 3σ level are shown as green mesh. The **8a**-, **8b**-, and **8c**-bound structures of CYP3A4 have the PDB ID codes 7UFB, 7UFC, and 7UFD, respectively.

Spectral changes induced by **8a**–**c** are shown in Figure 5A–C. All compounds produced type-II spectral shifts, and the binding affinity increased upon linker elongation (K_s_ of 0.120 μM for **8a** and 0.035 and 0.015 μM for **8b** and **8c**, respectively; Table 1). The elongated *S*/*S* conformer, **8c**, had the lowest IC_50_ (0.21 μM vs. 0.60 and 0.82 μM for **8a** and **8b**, respectively) and the highest stabilizing and heme-protective effects (compare ΔT_m_ and percentage of heme destroyed in Table 1 and Table 2). Relative to the Boc-containing compounds, attachment of the end-pyridine had a mixed effect. Compared to **4b**, a two-fold increase in K_s_ and two-fold decrease in IC_50_ were observed for **8a.** For **8b**, both parameters were moderately increased (by ~20%) relative to those for **4f**. In contrast, compared to **4h**, the binding affinity and inhibitory potency of **8c** were markedly improved (three-fold decrease in K_s_ and IC_50_). Interestingly, the replacement of Boc with pyridine had no significant effect on ΔT_m_ and H_2_O_2_-induced heme destruction. One notable feature of **8b** was the slower heme ligation rate: 5.6 s^−1^ vs. 10.3 and 15.0 s^−1^ for **8a** and **8c**, respectively (Table 2). However, the reaction remained biphasic for all bi-pyridine compounds. Structural information regarding **8a**–**c**-bound CYP3A4 helped to interpret these experimental findings.

#### 2.4.2. Crystal Structures of **8a**-, **8b**-, and **8c**-Bound CYP3A4

The X-ray structures of CYP3A4 complexed with **8a**, **8b**, and **8c** were solved to 2.25, 2.35, and 2.90 Å, respectively (Tables S1 and S2). The observed ligand orientations are shown in Figure 5D–F. Because **8a**–**c** were not fully symmetrical and differ in the linker carbonyl positions (Figure 2), it was possible to distinguish whether the ligating moiety was the head- or end-pyridine based on the electron density maps. Intriguingly, only **8b** coordinated with CYP3A4 via the head-group, while **8a** and **8c** ligated in the reverse orientation, with the head-pyridine in the substrate channel and the end-pyridine coordinated with the heme. However, the R_1_ group was still placed at the P1 site and R_2_ in the P2 area (Figure 5D–F). Generally, this distorted mode of side-group placement is unfavorable and has only been observed in inhibitors with *S*/*R* configuration [16,30].

**Figure 6:**
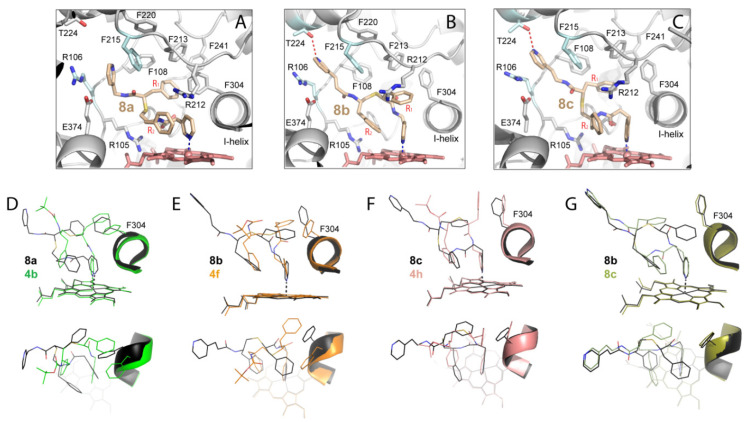
Interactions mediated by **8a**–**c** in the active site of CYP3A4 (**A**–**C**) and structural comparison of the **8a**–**4b** (**D**), **8b**–**4f** (**E**), **8c**–**4h** (**F**), and **8b**–**8c** pairs (**G**). (**A**–**C**) **8a** and **8c** bind in the reverse orientation, where the end-pyridine ligates to the heme and the head-group stacks in the substrate access channel, while the R_1_ and R_2_ side-groups (indicated) are still placed at the P1 and P2 sites, respectively. In all three inhibitors, the pyridine group bound in the substrate channel forms π–π and cation–π interactions with Phe215 and the guanidine group of Arg106, respectively. The H-bonds to Thr224 are established only by **8b** and **8c** (shown as red dotted lines). (**D**–**G**) The side and top views of structural overlays (upper and lower panels, respectively) highlight positional differences in the backbone and functional groups. The central part of the I-helix is depicted to demonstrate the adaptive rotameric changes in Phe304. The **4b**-, **4f**-, and **4h**-bound CYP3A4 structures used for comparison have the PDB ID codes 6DA3, 6DAC, and 6DAG, respectively.

Crystal structure examination showed that, due to the shorter pyridine spacer, **8a** could not strongly ligate to the heme, as evident from the longer Fe-N bond (2.29 Å vs. 2.04–2.10 Å for **8b**,**c**; Table 3). Additionally, in this subset of inhibitors, only **8c** could form an H-bond via the carbonyl oxygen to the active site Ser119. Thus, one advantage of the reverse orientation of **8c** was formation of this critical interaction with Ser119, strictly required for potent inhibition [27]. In **8a**, but not **8b**, the analogous carbonyl was positioned suitably for a long-range polar interaction with the Arg105 guanidine (3.8 Å away), which could strengthen the inhibitory complex, to some extent. The end moieties of **8a**–**c** bound in the substrate channel (Figure 6A–C), where the pyridine rings were sandwiched between the Phe215 and Arg106 side groups, forming π–π and cation–π interactions, respectively. The shorter **8a** could not fully maximize these interactions, although its reverse orientation seemed to provide some assistance, as the entire molecule rotated and tilted toward the channel to bring the pyridine moiety closer to Phe215 and Arg106. Another consequence of this reorientation was the R_2_ shift toward the heme-ligating pyridine. This increased the overlap between the aromatic rings, but eliminated cation–π interaction with Arg105, which became too distant. The latter interaction was also lost for **8c** due to its similar core rotation. Importantly, only elongated **8b** and **8c** could H-bond to the Thr224 hydroxyl group via the end-pyridine N heteroatom (Figure 6B,C). The **8c**-mediated interaction was much stronger (H-bond length of 2.93 vs. 3.29 Å for **8b**), which could be another benefit of the reverse orientation.

Pairwise comparison of bi-pyridine and Boc-containing counterparts is shown in Figure 6D–G. The most drastic changes in the ligand-binding mode caused by the end-group attachment were observed for the **8a**–**4b** pair (Figure 6D). To place the tail-group inside the substrate channel, the heme-ligating pyridine of **8a** rotated by 30° and, as the backbone curvature changed, the entire molecule shifted away from the I-helix, tilted toward the substrate channel, and merged closer to the heme. However, this did not significantly alter the Fe–N bond (2.29 Å vs. 2.26 Å in **4b**). Thus, the higher inhibitory potency of **8a** might have been due to additional interactions established in the substrate channel and stronger aromatic contacts at the P2 site. On the other hand, the lack of H-bonding to Ser119 and cation–π interactions with Arg105 along with weaker hydrophobic interactions at the P1 site could be the reason behind the decrease in the binding affinity of **8a**.

A similar trend was observed for the **8b**–**4f** pair (Figure 6E). For **8b**, the association of the end-pyridine in the substrate channel led to the backbone straightening and, subsequently, rearrangements at the P1 site. However, due to the longer R_1_-pyridyl spacer, the overall conformational changes were not as drastic as those in **8a**. The most notable change was in R_1_-phenyl, which, after pulling away from the P1 pocket, swung aside to form T-shaped π-stacking with Phe304 and the heme-ligating pyridine (Figure 6B). Despite the shorter Fe–N bond in **8b** (2.10 Å vs. 2.29 Å in **4f**), there was no improvement in the binding and inhibitory strength, possibly due to the loss of H-bonding to Ser119 and R_1_-mediated hydrophobic contacts at the P1 site.

Comparison of the **8c**–**4h** pair (Figure 6F) showed that **8c** shifted toward the substrate channel and tilted closer to the heme, similar to **8a**. Likewise, the **8c** R_1_-phenyl pulled away from the P1 pocket, whereas R_2_ moved deeper into the P2 site, further from Arg105, and closer to the heme-ligating pyridine. The impact of the heme-ligating group reversal can be more clearly seen when **8c** and **8b** are compared (Figure 6G). The tail-moieties of these compounds nearly coincided, but marked differences in the R_1_ and R_2_ positioning were evident. The **8c** conformation appeared to be more favorable because (i) more extensive aromatic and hydrophobic contacts were established at the P2 site due to the deeper protrusion of R_2_-phenyl, and (ii) R_1_-phenyl was closer to the hydrophobic cluster adjacent to the I-helix and could form contacts with multiple residues (Phe108, Ile120, Phe213, Ile301, Phe304, and Phe241). As mentioned, the **8b** R_1_-phenyl was at the center of the active site cavity and its stacking interactions were limited to Phe304 and the heme-ligating pyridine (Figure 6B). These distinctions, along with the stronger H-bonding of **8c** to Ser119 and Thr224, could largely account for its improved K_s_ and IC_50_ (Table 1). That, in this sub-group, only **8c** was superior to the Boc-containing counterpart showed that a small, chemically simple R_3_-group, such as pyridine, did not strongly contribute to the binding and inhibitory strength, but could modulate these parameters through the interplay with other structural determinants that defined the ligand-binding mode. The distinct orientations of **8b** and **8c**, in turn, emphasized the importance of side-group configuration and further demonstrated the remarkable ability of CYP3A4 to select and mold ligands in the active site for optimization of protein–ligand interactions.

### 2.5. Interaction of CYP3A4 with ***10a*** and ***10b***

#### 2.5.1. Spectral, Kinetic, and Inhibitory Properties of **10a**–**b**

The last two compounds in this series, **10a** and **10b**, had the head-group linked via the most optimal propyl spacer [17] and the end-pyridine attached through a single or double amide bond (Figure 2). Both analogues were strong type-II binders and had a comparable binding affinity for CYP3A4, the highest for this series (K_s_ of 0.008–0.009 μM). It was shown that **10a** had the highest inhibitory potency as well: IC_50_ of 0.15 μM vs. 0.21 μM for **10b**. Both compounds ligated to CYP3A4 with a similar rate and protected the heme from hydrogen peroxide more effectively than other analogues (Table 2). Overall, **10a** was the series’ lead compound, but, relative to the Boc-containing **3a**, a notable improvement was only observed in the binding affinity (two-fold increase) and heme protection (5% increase). For this sub-group, the attachment of R_3_-pyridine had virtually no effect on IC_50_ and slightly lowered thermostability of CYP3A4 (ΔT_m_ of 7.2–7.3 °C vs. 8.1 °C for **3a**). As with the other analogs, determination of the crystal structures of **10a**/**b**-bound CYP3A4 helped to better understand the experimental results.

#### 2.5.2. Crystal Structures of **10a**- and **10b**-Bound CYP3A4

**10a** and **10b** co-crystallized with the wild-type CYP3A4 in the I222 space group, with one loosely packed molecule per asymmetric unit. Similar to other pyridyl–propyl containing analogues [17], the binding modes of **10a**,**b** could not be accurately defined in this crystal form due to high thermal motion and discontinuous electron density maps. To overcome this problem, we used the same strategy and co-crystallized **10a**,**b** with the CYP3A4 variant containing two surface mutations, K421A and K424A. This variant preferably crystallized in the C2 space group, where one of the two molecules present in the asymmetric unit was well defined. As seen in Figure S2, there were only minor differences in the ligand-binding mode imposed by distinct crystal packing. The structures of **10a**- and **10b**-bound CYP3A4 were determined to 2.4 and 2.7 Å resolution, respectively (Table S2). The best-defined molecules from each structure (molecules A) were used for structural and comparative analysis.

**Figure 7:**
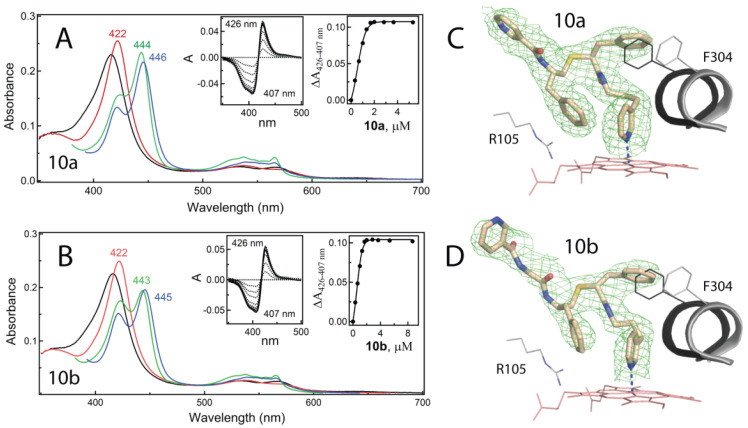
Spectral properties (**A**,**B**) and the binding modes of **10a**,**b** (**C**,**D**). (**A**,**B**) Ligand-induced spectral changes in CYP3A4. Absorbance spectra of ferric ligand-free and inhibitor-bound CYP3A4 are in black and red, respectively. Spectra of ferrous ligand-bound CYP3A4 and its CO-adduct are in green and blue, respectively. Left insets are the difference spectra recorded during equilibrium titrations; the right insets are titration plots with quadratic fittings. The derived K_s_ values are listed in Table 1. (**C**,**D**) Binding modes of **10a**–**b** observed in the crystal structures. The central part of the I-helix and Phe304 in the inhibitory complexes and water-bound CYP3A4 (5VCC structure) are depicted in gray and black, respectively. Polder omit electron density maps contoured at 3σ level are shown as green mesh. The **10a**- and **10b**-bound structures of CYP3A4 have the PDB ID codes 7UFE and 7UFF, respectively.

**Figure 8:**
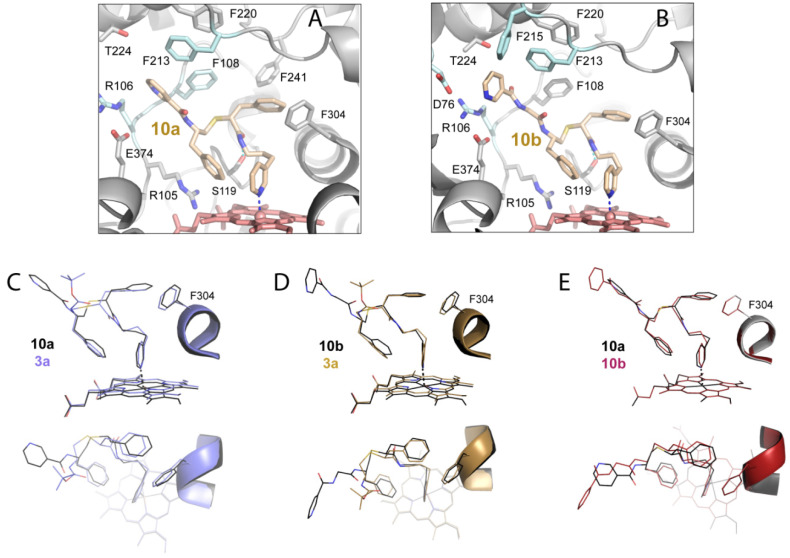
Interactions mediated by **10a**–**b** in the CYP3A4 active site (**A**,**B**) and comparison of the **10a**–**3a** I, **10b**–**3a** (**D**), and **10a**–**10b** pairs (**E**). (**A**,**B**) **10a** and **10b** bind in a traditional orientation and place the end-pyridine into the substrate access channel. Because of the longer R_2_–R_3_ linker in **10b**, its end-pyridine inserts further into the channel and cannot orient optimally for π–π and cation–π interactions with Phe215 and Arg106. Among series-VI inhibitors, only **10a**,**b** formed S–π interactions with Phe108 via the backbone sulfur atom. (**C**–**E**) The side and top views of structural overlays (upper and lower panels, respectively) show only small positional differences in the backbone and side-group orientations caused by R_3_-pyridine attachment. No notable changes in Phe304 were observed as well. The CYP3A4-**3a** complex structure used for comparison has the PDB ID code 7KVH.

Both compounds bound to CYP3A4 in a traditional orientation, distorted the I-helix to the same extent (Figure 7C–E), and formed strong H-bonds to Ser119. As expected, differences in the length and flexibility of the R_2_–R_3_ linker led to a distinct end-pyridine placement. In **10a**, the shorter tether disallowed the parallel stacking of the pyridine ring with the nearby Phe108 and Phe213. As a result, only partially overlapping T-shaped stacking with Phe108 could be established (Figure 8A). The R_3_-pyridine barely overlapped with the Arg106 guanidine as well, due to which cation–π interactions were also weakened. In **10b**, the end moiety was on a longer tether and inserted further into the substrate channel (Figure 7B). This also precluded the optimal π–π stacking with the neighboring Phe213 and Phe215. Moreover, the end-pyridine in **10b** was perpendicular, rather than parallel, to the Arg106 guanidine. This diminished the cation–π interactions, but enabled polar interactions with the nearby Asp76 (3.6 Å away). Neither compound could H-bond to Thr224, but both formed strong H-bonds to Ser119 (Table 3). That **10a** has a higher inhibitory potency could be due to the stronger heme coordination (Fe–N bond of 2.16 Å vs. 2.21 Å for **10b**) and more favorable interactions mediated by R_3_.

Compared to **8a**–**c**, the R_3_-pyridines of **10a**,**b** oriented less optimally due to the shorter (in **10a**) or more rigid (in **10b**) R_2_–R_3_ linkage. One distinctive feature that provided additional stabilization of the inhibitory complexes with **10a**,**b** was the S–π interaction between the backbone sulfur atom and Phe108 ring (only 3.7 Å away). This interaction was not observed in **8a**–**c**-bound structures, but was established by **3a** and other pyridyl–propyl-containing analogues. Comparison of the **10a**–**3a**, **10b**–**3a,** and **10a**–**10b** pairs (Figure 8C–E) showed that the attachment of R_3_-pyridine and elongation of the R_2_–R_3_ linker caused only minor adjustments in the inhibitor core and side-group positioning. That **10a** was a tighter binder but not a stronger inhibitor than **3a** suggested, once again, that a small, chemically simple R_3_-group could improve the binding strength by establishing additional contacts with the protein residues, but could not feasibly diminish CYP3A4 activity due to its inability to effectively block the substrate channel and prevent access to the active site.

## 3. Discussion

In a continuing effort to identify structural attributes required for the potent inhibition of CYP3A4, the major human drug-metabolizing enzyme, we designed eight new ritonavir-like inhibitors differing in the head- and end-groups and their connecting fragments (Figure 2). One unresolved question that we addressed was the degree of importance of the flexibility of the head-group linker. Our previous study showed that the pyridyl–propyl spacer, which resulted in a seven-atom separation between the head- and R_1_-group, was the most optimal for the binding and inhibition of CYP3A4 [17]. At the same time, according to the structural data, the flexible propyl connector increased the inhibitor’s mobility in the active site. Therefore, we synthesized compound **4** with the same seven-atom pyridine–R_1_ separation, but replaced the flexible propyl chain with a rigid peptide bond (Figure 2). This structural change had a profound negative impact: the binding affinity and inhibitory potency of **4** were decreased by 100- and 10-fold, respectively, and its stabilizing and heme-protective effects were largely diminished (Table 1 and Table 2). It should be noted that, under our assay conditions, IC_50_ was limited to 0.050 μM (0.1 μM CYP3A4; 1:1 inhibitory complex), whereas the dissociation constant does not depend on the protein concentration. This could explain the lower spread in IC_50_ and disparity between the K_s_ and IC_50_ values. Another manifestation of the low affinity of the CYP3A4-**4** complex was its dissociation during crystallization. A similar phenomenon was observed only with series-V compound **6**, which had eight-atom pyridine–R_1_ separation [17]. Thus, sufficient length/flexibility of the head-group spacer is a prerequisite for strong heme coordination.

Another factor that affects the heme coordination strength is the pyridine nitrogen position. Spectral and kinetic data on **5a**/**b**-bound CYP3A4 show that switching from *meta*- to *para*-N-pyridine facilitates inhibitor association and leads to a stronger heme ligation, as evidenced by the largest Soret shift and the highest ΔA_max_ observed for **5b** (Table 1 and Table 2). However, compared to the *meta*-N-containing counterparts, the most notable improvements in K_s_, IC_50_, thermostability, and heme protection were observed for the shorter analogue, **5a** (Table 1 and Table 2). This shows that regulation of the inhibitory strength is complex and multifactorial, and that stereoelectronic requirements for optimal heme ligation play a key role. Spatial relationships in the donor–acceptor electronic structures are known to be important. Our data demonstrate that steric constraints imposed on the tethered heterocycle in ritonavir-like compounds can be increased or relaxed, to some extent, by changing the length/flexibility of the tether and/or position of the electron-donating N-heteroatom.

Bi-pyridine compounds were designed to test if/how R_3_-pyridine, attached via various linkers, affects the ligand binding kinetics, affinity, and inhibitory strength. Surprisingly, no notable changes were observed in the ligand binding kinetics, which remained biphasic, with comparable rate constants and fast phase percentages (Table 2). It was suggested earlier that the biphasicity of the heme ligation reaction might stem from the ability of ritonavir-like molecules to enter the active site either with the head- or end-moiety first [24,25,29]. Structural data presented here provide direct evidence for this and, more importantly, show that the end-pyridine can ligate to the heme as well (Figure 5D–F and Figure 6A–C). The reverse coordination was preferable when the inhibitor had a suboptimal head-group spacer (**8a**) or side-group configuration (**8c**). For **8c** (*S*, *S*), the end-pyridine ligation had two advantages: it allowed H-bonding to Ser119, which was impossible for **8b** (*R*, *S*), and strengthened H-bonding to Thr224 (Table 3). As a result, the binding affinity and inhibitory potency of **8c**, as well as its ligation rate and heme protective ability, were markedly enhanced (Table 1 and Table 2). The reverse orientation of **8b**,**c** further supports the notion that the relationship between stereochemistry and preferential orientation in the CYP3A4 active site is not straightforward [30] and, as this study demonstrates, can be modulated by the head- and end-group linkers. That the binding of bi-pyridine compounds remained biphasic could be the consequence of (i) intrinsic heterogeneity and nonspecific aggregation of recombinant CYP3A4 [33,34], (ii) slow ligand reorientation inside the active site, and/or (iii) ligand dissociation and re-entrance.

**Figure 9:**
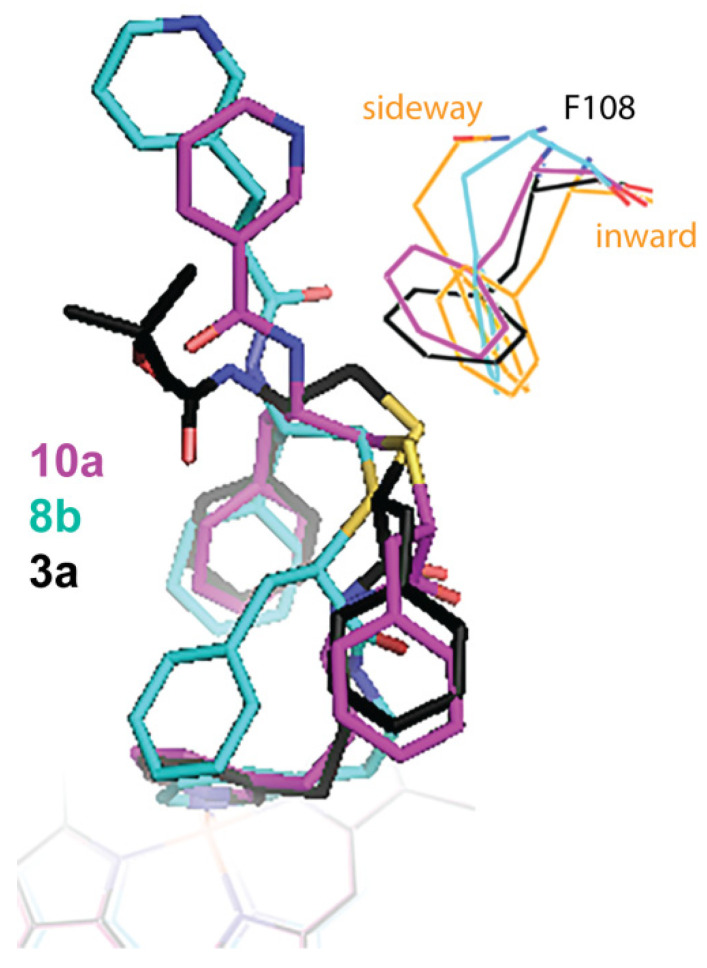
S–π interaction between the inhibitor backbone sulfur atom and Phe108. Only compounds with the elongated pyridyl–propyl head-linker, such as **10a**,**b** and **3a**, but not **8b**, are positioned suitably for the S–π interaction between the backbone sulfur atom and the aromatic ring of Phe108. Due to intrinsic conformational heterogeneity of the Arg106-Pro110 fragment, Phe108 adopts a ‘sideways’ or ‘inward’ orientation (in orange; ligand-free 5VCC structure), which can be readjusted to optimize the aromatic overlap with the S-heteroatom. In **10b**, the backbone and Phe108 positions are similar to those in **10a** and are not shown for clarity.

As anticipated, among bi-pyridine compounds, the elongated **10a**,**b** analogues were superior to **8a**–**c**. Moreover, **10a** was the series’ lead compound, whose binding affinity exceeded and the inhibitory potency matched those of ritonavir (K_s_ and IC_50_ for ritonavir were 0.019 μM and 0.13 μM, respectively, under our experimental conditions). The common features of **10a**,**b** include a strong H-bond to Ser119 and S–π interaction between the backbone sulfur atom and Phe108 aromatic ring (Figure 8A,B). S–π interactions, known to play an important role in protein folding and stabilization [35], were not evaluated in series-V compounds [17]. In CYP3A4, the Arg106-Pro110 fragment is part of the B–C loop, lining the wall of the catalytic cavity, and can adopt two conformations, with the Phe108 side chain in a ‘sideways’ or ‘inward’ orientation [36]. As shown in Figure 9, Phe108 with **8b** is in a ‘sideways’ orientation, with no S–π overlap. In the elongated **10a**,**b** and **3a**, the backbone raises higher and closer to the ‘inward’ Phe108, which centers above the S-heteroatom to maximize overlap. Thus, the inhibitory complex can be stabilized via a concerted movement of the Arg106–Pro110 fragment, Phe304 side chain, and F–G loop residues. Importantly, only some, but not all, potent inhibitors can establish the Phe108-mediated S–π interaction (e.g., **3i**; IC_50_ of 0.15 μM; 7KVS structure [17]). Thus, this protein-ligand interaction is beneficial, but not a prerequisite for the potent inhibition of CYP3A4.

The equal potency of **10a** and **3a** indicates, in turn, that a small end-group, such as pyridine, cannot effectively block the substrate channel to further diminish catalytic activity. Thus, one optimization strategy would be the R_3_ enlargement and branching. As evident from the properties of cobicistat, this approach could decrease the off-target activities of the newly designed inhibitors and improve their selectivity for CYP3A4. Cobicistat is a close derivative of ritonavir [6], with only two distinctive features (Figure 1): it lacks the central backbone hydroxyl group, critical for the binding to HIV-1 protease, and has a valine-to-morpholine substitution in the end-moiety. As a result, cobicistat is devoid of undesired anti-HIV activity and, compared to ritonavir, has improved physicochemical properties and fewer side effects and off-target activities. Importantly, even though both these pharmacoenhancers cross-react with other CYPs, their inhibitory potency for CYP3A4 is >200-fold higher [13]. Our analogues lack the backbone hydroxyl as well, but contain much smaller/chemically simpler end-groups. Therefore, they are not expected to possess antiviral activity or higher selectivity for CYP3A4. Further, due to structural similarity and substantially overlapping substrate specificities between the major CYP3A isoforms, 3A4 and 3A5, ritonavir and cobicistat are considered as CYP3A inhibitors. Likewise, the inhibitory potency of selected series-VI analogues tested against recombinant CYP3A5 was found to be similar, with the IC_50_ estimates for **5b**, **8c**, and **10a** being within the 180–200 nM range vs. 300 nM for ritonavir. Because the architecture of the CYP3A4/5 active sites differ, particularly in the upper wall formed by flexible F’- and G’-helices and the connecting loop [37,38], it might be possible to design inhibitors that would be less or more specific for CYP3A5 through the enlargement, branching, and rigidification of the end-moiety.

Lastly, we intentionally limited the scope of this study by including compounds only with phenyl side-groups to enable comparisons with the previously reported Boc-containing analogues and to simplify data analysis and interpretation. As demonstrated earlier [16,17,26,28,29,30], the side-group size, hydrophobicity/aromaticity, and stereo configuration are the major factors that contribute to the binding and inhibitory strength. The phenyl group is the largest R_1_ substituent that can optimally fit into the restricted P1 pocket. In contrast, the P2 site is more spacious and can easily accommodate larger R_2_ moieties, such as indole and naphthalene [17,30]. While the coordination of the heme-ligating pyridine drives the inhibitory complex formation, the overall binding mode is predominantly defined by the R_1_/R_2_-mediated interactions, which, as this and previous studies showed, can be modulated through backbone spacing. Importantly, the side-group chemical nature and stereo configuration seem to control the binding orientation and inhibitory potential regardless of whether the linkage between the functional groups is optimal or not. This was demonstrated with series-IV and V analogues [17,30], where a single R_2_-phenyl-to-indole/naphthalene substitution strengthened the heme coordination and markedly improved K_s_ and IC_50_. Hence, it would be worthwhile to further test the relative importance of side-group functionalities and steric constraints imposed on the heme-ligating group using compounds with R_2_-indole/naphthalene.

In conclusion, spectral, functional, and structural data on series-VI analogues demonstrated the multifactorial regulation of inhibitory strength and identified steric constraints imposed on the tethered heme-ligating group as a key impact factor. These constraints must be minimized to improve the inhibitory potency for CYP3A4, which can be achieved by increasing length/flexibility of the head-group linker, switching from *meta*- to *para*-position in the heme-ligating N-pyridine, and changing side-group configuration. The impact of the R_3_-pyridine attachment was not uniform and depended on the head/end-group spacers and side-group stereochemistry. This interplay between pharmacophoric determinants along with the end-group enlargement can be used to develop strategies for further inhibitor optimization.

## 4. Materials and Methods

*General Chemistry Methods*—^1^H NMR spectra were recorded on Bruker DRX 400 MHz, Bruker DRX 500 MHz, or Bruker Avance 600 MHz spectrometers. Chemical shifts (δ) were reported in ppm for the solution of the compound in CD_3_OD or CDCl_3_ (with internal reference TMS), and J values in hertz. NMR data were processed using TopSpin 3.5 software, and ACD/Spectrus Processor 2018.2.5. LRMS and HRMS data were obtained via ESI LC-TOF on a Waters (Micromass) LCT Premier spectrometer (Waters), with PEG as the calibrant for HRMS. The optical rotation was recorded on a Rudolph Autopol III Automatic Polarimeter at room temperature in MeOH. The purity of the final products was verified by NMR with TMS as a standard. TLC was performed using EMD Millipore silica gel 60 F_254_ aluminum plates. Separation by column chromatography was performed using Fisher silica gel 60 (230–400 mesh). All reactions were performed with commercially available reagents (Aldrich, Thermo-Fisher, Alfa Aesar, Acros, Oakwood, and Millipore). Anhydrous solvents were acquired using a solvent purification system (Inert PureSolv and JC Meyer systems) or purified according to standard procedures.

### 4.1. Synthesis of Analogues

Compound **1** was prepared as described previously [39,40,41], with Boc-protection and tosylation of commercially available L-phenylalaniol. Compounds **2a** and **2b** were also prepared as described previously [39,40,41], with either *D-* or *L-*α-thio-phenylalanine, respectively [42].

### 4.2. Synthesis of Compound ***3***

*N*-Boc-glycine (1.00 g, 5.71 mmol) was dissolved in DMF (15 mL). To this solution, 3-amino pyridine (0.59 g, 6.22 mmol, 1.1 eq) was added, along with EDAC (1.31g, 6.84 mmol, 1.2 eq), HOBt (1.05 g, 6.84 mmol, 1.2 eq), and DIPEA (2.21, 17.1 mmol, 3 eq). The reaction was stirred at room temperature overnight. Upon completion, the solvent was evaporated, and the reaction mixture was diluted with ethyl acetate. The organic layer was then washed with saturated NaHCO_3_, water, and brine. The combined organic layers were dried over MgSO_4_ and concentrated in vacuo to afford the Boc-protected 2-amino-*N*-(pyridine-3-yl)acetamide (1.08 g, 75%). The Boc product was dissolved in 12 mL of DCM and placed in an ice bath. An amount of 4 mL of TFA was added dropwise, and the solution was allowed to come to room temperature over 3 h. Upon completion, the solvent was evaporated, and the reaction mixture was neutralized with saturated NaHCO_3_. The basic solution was washed with DCM and concentrated in vacuo. The salts were filtered and washed with EtOH and acetone, and the filtrate was evaporated to afford the crude product, **3**, as an opaque oil, which was used for the synthesis of **4** without further purification. LRMS was *m/z* calculated for C_7_H_10_N_3_O [M + H]^+^: 152.1. Found: 152.1.

### 4.3. Synthesis of Compound ***4***

**Scheme 1:**
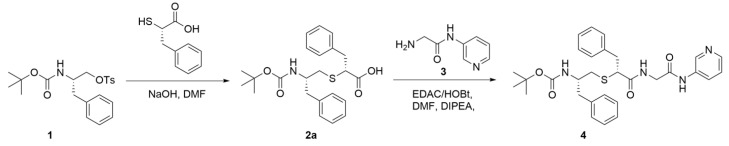
Synthesis of compound **4**.

Synthesis of compound **4** is outlined in Scheme 1. Crude **3** (0.54 g, 3.6 mmol, 6 eq) was dissolved in DMF (5 mL) and added to crude **2a** (0.25 g, 0.6 mmol). To this solution, EDAC (0.17 g, 0.9 mmol, 1.5 eq) and HOBt (0.14 g, 0.9 mmol, 1.5 eq) were added, followed by the addition of DIPEA (0.23 g, 1.8 mmol, 3 eq). The reaction was stirred at room temperature overnight. Upon completion, the solvent was evaporated and the reaction mixture was diluted with ethyl acetate. The organic layer was then washed with saturated NaHCO_3_, water, and brine. The combined organic layers were dried over MgSO_4_ and concentrated in vacuo to produce the crude product, which was purified via column chromatography (95:5 EtOAc:MeOH). The pure product **4** was obtained as a white fluffy solid (0.054 g, 16%). TLC: EtOAc/MeOH 90:10 (Rf. 0.41). ^1^H NMR (400 MHz, CDCl_3_) δ 9.11 (s, 1H (NH)), 8.61 (s, 1H), 8.33 (s, 1H), 8.06 (d, *J* = 7.0 Hz, 1H), 7.55 (s, 1H), 7.34–7.07 (m, 10H), 4.91 (s, 1H), 4.10 (dd, *J* = 5.1, 17.1 Hz, 1H), 3.96 (dd, *J* = 5.2, 16.4 Hz, 1H), 3.72 (m, 1H), 3.23 (dd, *J* = 8.0, 13.5 Hz, 1H), 2.99 (dd, *J* = 7.0, 13.8 Hz, 1H), 2.79 (m, 2H), 2.60 (dd, *J* = 7.3, 13.7 Hz, 1H), 2.48 (bs, 1H), and 1.40 (s, 9H). HRMS *m/z* calculated for C_30_H_37_N_4_O_4_S [M + H]^+^: 549.2535. Found: 549.2512.

### 4.4. Synthesis of Compounds ***5a*** and ***5b***

**Scheme 2:**
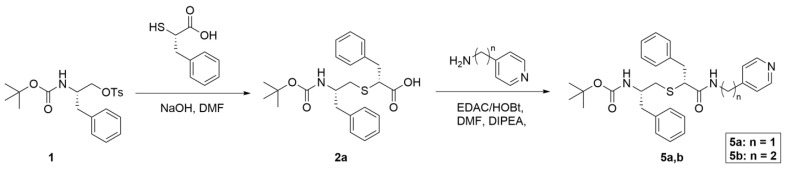
Synthesis of compounds **5a** and **5b**.

Synthesis of **5a**,**b** is outlined in Scheme 2. Crude **2a** (0.12 g, 0.29 mmol) was dissolved in DMF (4 mL). To this solution, EDAC (0.084 g, 0.44 mmol, 1.5 eq) and HOBt (0.067 g, 0.44 mmol, 1.5 eq) were added, followed by the addition of 4-(2-aminoethyl)pyridine (0.054 g, 0.44 mmol, 1.5 eq) and DIPEA (0.11 g, 0.87 mmol, 3 eq). The reaction was stirred at room temperature overnight. Upon completion, the solvent was evaporated and the reaction mixture was diluted with ethyl acetate. The organic layer was then washed with saturated NaHCO_3_, water, and brine. The combined organic layers were dried over MgSO_4_ and concentrated in vacuo to produce the crude product, which was purified via column chromatography (95:5 EtOAc:MeOH). The pure product **5a** was obtained as an off-white fluffy solid (0.029 g, 19%). TLC: EtOAc/MeOH 90:10 (Rf. 0.52). ^1^H NMR (400 MHz, CDCl_3_) δ 8.44 (d, *J* = 5.9 Hz, 2H), 7.33–7.17 (m, 8H), 7.12 (d, *J* = 6.8 Hz, 2H), 6.94 (d, *J* = 5.9 Hz, 2H), 6.50 (t, *J* = 7.3 Hz, 1H (NH)), 4.55 (d, *J* = 9.3 Hz, 1H (NH)), 3.92 (q, *J* = 6.5 Hz, 1H), 3.51 (m, *J* = 6.9 Hz, 1H), 3.43 (t, *J* = 7.1 Hz, 1H), 3.35 (m, *J* = 6.7 Hz, 1H), 3.25 (dd, *J* = 7.4, 13.7 Hz, 1H), 2.93 (dd, *J* = 6.8, 13.7 Hz, 1H), 2.76 (d, *J* = 7.0 Hz, 2H), 2.68 (t, *J* = 7.0 Hz, 2H), 2.62 (dd, *J* = 5.5, 13.9 Hz, 1H), 2.51 (dd, *J* = 5.7, 13.5 Hz, 1H), and 1.40 (s, 9H). HRMS *m/z* calculated for C_30_H_37_N_3_O_3_SNa [M + Na]^+^: 542.2454. Found: 542.2426. Compound **2a** and 3-(4-pyridyl)propylamine produced the pure product **5b** as a white, fluffy solid (0.033 g, 20%). TLC: EtOAc/MeOH 90:10 (Rf. 0.48). ^1^H NMR (400 MHz, CDCl_3_) δ 8.47 (d, *J* = 5.9 Hz, 2H), 7.30–7.16 (m, 8H), 7.13 (d, *J* = 6.8 Hz, 2H), 7.04 (d, *J* = 6.2 Hz, 2H), 6.52 (t, *J* = 6.2 Hz, 1H (NH)), 4.60 (d, *J* = 7.3 Hz, 1H (NH)), 3.95 (q, *J* = 5.8 Hz, 1H), 3.48 (t, *J* = 7.0 Hz, 1H), 3.25 (m, 2H), 3.16 (quint, *J* = 6.9 Hz, 1H), 2.96 (dd, *J* = 6.9, 13.7 Hz, 1H), 2.79 (d, *J* = 6.8 Hz, 2H), 2.66 (m, 1H), 2.58 (dd, *J* = 5.9, 13.5 Hz, 1H), 2.48 (t, *J* = 7.7 Hz, 2H), 1.70 (quint, *J* = 7.1 Hz, 2H), and 1.40 (s, 9H). HRMS *m/z* calculated for C_31_H_40_N_3_O_3_S [M + H]^+^: 534.2791. Found: 534.2766.

### 4.5. Synthesis of Compounds ***7a***–***c***

Compound **6a** (0.25 g, 0.50 mmol) was prepared as described previously [39], dissolved in 4.5 mL DCM, and placed in an ice bath. An amount of 1.5 mL of TFA was added dropwise and the solution was stirred at room temperature for 2 h. Upon completion, the volatiles were evaporated. The residue was neutralized with saturated NaHCO_3_ and extracted with DCM (3x excess). The combined DCM was washed with brine, dried over MgSO_4_, and concentrated in vacuo to produce the crude yellow oily product **7a**, which was used in the next step without further purification. LRMS *m/z* calculated for C_24_H_29_N_3_OS [M + H]^+^: 406.2. Found: 406.2. Compound **6b** produced crude **7b** (yellow oil) after stirring in 25% TFA at room temperature overnight. HRMS *m/z* calculated for C_25_H_30_N_3_OS [M + H]^+^: 420.2110. Found: 420.2109. Compound **6c** produced crude **7c** (yellow oil) after stirring in 1:1 TFA:DCM for 3 h at room temperature. LRMS *m/z* calculated for C_25_H_30_N_3_OS [M + H]^+^: 420.2. Found: 420.3.

### 4.6. Synthesis of Compounds ***8a***–***c***

**Scheme 3:**
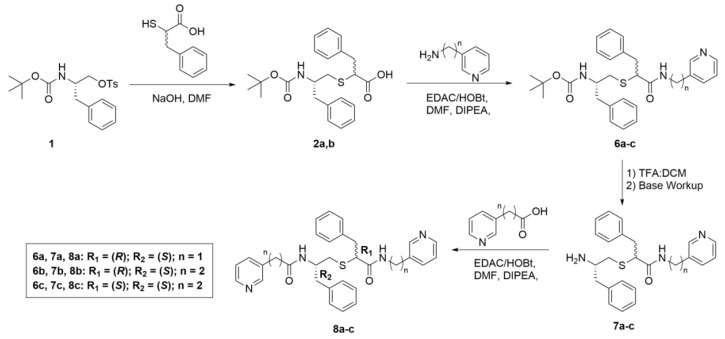
Converged synthesis of **8a**–**c**.

Synthesis of **8a***–***c** is outlined in Scheme 3. Crude **7a** (0.2 g, 0.49 mmol) was dissolved in DMF (5 mL). To this solution, 3-pyridylacetic acid hydrochloride (0.09 g, 0.49 mmol, 1 eq), EDAC (0.14 g, 0.74 mmol, 1.5 eq), and HOBt (0.11 g, 0.74 mmol, 1.5 eq) were added, followed by the addition of DIPEA (0.19 g, 1.47 mmol, 3 eq). The reaction was stirred at room temperature overnight. Upon completion, the solvent was evaporated, and the reaction mixture was diluted with ethyl acetate. The organic layer was then washed with saturated NaHCO_3_, water, and brine. The combined organic layers were dried over MgSO_4_ and concentrated in vacuo to produce the crude product, which was purified via column chromatography (90:10 DCM:MeOH). The pure product **8a** was obtained as a white fluffy solid (0.12 g, 47%). TLC: EtOAc/MeOH 90:10 (Rf. 0.15). ^1^H NMR (400 MHz, CDCl_3_) δ 8.45 (dd, *J =* 4.8, 11.6 Hz, 2H), 8.36 (bd, *J =* 10.5 Hz, 2H), 7.47 (d, *J =* 7.8 Hz, 1H), 7.33 (d, *J =* 7.9 Hz, 1H), 7.27–7.13 (m, 9H), 6.99 (m, 3H), 6.00 (d, *J =* 8.2 Hz, 1H (NH)), 4.40 (dd, *J =* 6.2, 15.1 Hz, 1H), 4.23 (m, 2H), 3.50 (t, *J =* 7.4 Hz, 1H), 3.41 (s, 2H), 3.21 (dd, *J =* 8.1, 13.8 Hz, 1H), 2.93 (dd, *J =* 6.6, 13.7 Hz, 1H), 2.80 (dd, *J =* 6.4, 13.8 Hz, 1H), 2.70 (quint, *J =* 7.3 Hz, 2H), and 2.60 (dd, *J =* 6.6, 13.6 Hz, 1H). HRMS *m/z* calculated for C_31_H_32_N_4_O_2_Sna [M + Na]^+^: 547.2144. Found: 547.2135. Compound **7b** and 3-pyridinepropinoic acid produced the pure product **8b** as a white fluffy solid (0.031 g, 29%). TLC: EtOAc/MeOH 90:10 (Rf. 0.15). ^1^H NMR (400 MHz, CDCl_3_) δ 8.39 (m, 3H), 8.28 (s, 1H), 7.49 (d, *J =* 7.7 Hz, 1H), 7.39 (d, *J =* 7.7 Hz, 1H), 7.27–7.13 (m, 10H), 7.06 (d, *J =* 7.7 Hz, 2H), 6.62 (t, *J =* 5.4 Hz, 1H (NH)), 6.03 (d, *J =* 8.6 Hz, 1H (NH)), 4.25 (q, *J =* 6.5 Hz, 1H), 3.50 (m, 1H), 3.33 (m, 2H), 3.17 (dd, *J =* 7.3, 13.7 Hz, 1H), 2.95 (m, 1H), 2.88 (m, 2H), 2.72 (m, 4H), 2.53 (dd, *J =* 5.1, 13.3 Hz, 1H), 2.45 (t, *J =* 6.4 Hz, 1H), and 2.38 (m, 2H). HRMS *m/z* calculated for C_33_H_36_N_4_O_2_Sna [M + Na]^+^: 575.2457. Found: 575.2450. Compound **7c** and 3-pyridinepropinoic acid afforded the pure product **8c** as a white crystalline solid (0.22 g, 66%). TLC: EtOAc/MeOH 90:10 (Rf. 0.16). ^1^H NMR (400 MHz, CDCl_3_) δ 8.38 (m, 3H), 8.33 (s, 1H), 7.42 (ddt, *J =* 15.0, 7.9, 1.9, 1.9 Hz, 2H), 7.29–7.14 (m, 10H), 7.06 (m, 1H), 7.02 (m, 2H), 6.28 (d, *J* = 8.3 Hz, 1H (NH)), 4.14 (sep, *J* = 7.1 Hz, 1H) 3.46 (m, 3H), 3.23 (dd, *J =* 7.5, 13.9 Hz, 1H), 2.86 (q, *J* = 7.1 Hz, 3H), 2.78 (m, 2H), 2.67 (td, *J =* 7.4, 14.7 Hz, 1H), 2.43 (m, 3H), and 2.34 (dd, *J =* 6.2, 13.7 Hz, 1H). HRMS *m/z* calculated for C_33_H_36_N_4_O_2_Sna [M + Na]^+^: 575.2457. Found: 575.2462.

### 4.7. Synthesis of Compounds ***10a*** and ***10b***

**Scheme 4:**
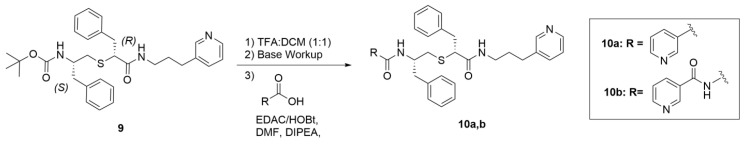
Synthesis of compounds **10a** and **10b**.

Synthesis of **10a**,**b** is outlined in Scheme 4. Compound **9** (0.1 g, 0.19 mmol), which was prepared as described previously for the series-V compound **3a [17]**, was dissolved in 2 mL DCM and placed in an ice bath. An amount of 2 mL of TFA was added dropwise and the solution was stirred at room temperature for 2 h. Upon completion, the volatiles were evaporated. The residue was neutralized with saturated NaHCO_3_ and extracted with DCM (3x excess). The combined DCM was washed with brine, dried over MgSO_4_, and concentrated in vacuo to produce the crude Boc-deprotected propyl-pyridyl thioether as a yellow oil, which was used in the next step without further purification. LRMS *m/z* calculated for C_26_H_32_N_3_OS [M + H]^+^: 434.2. Found: 434.2.

The crude Boc-deprotected propyl-pyridyl thioether (0.1 g, 0.23 mmol) was dissolved in DMF (3 mL). To this solution, EDAC (0.066 g, 0.35 mmol, 1.5 eq) and HOBt (0.053 g, 0.35 mmol, 1.5 eq) were added, followed by the addition of nicotinic acid (0.041 g, 0.33 mmol, 1.45 eq) and DIPEA (0.09 g, 0.7 mmol, 3 eq). The reaction was stirred at room temperature overnight. Upon completion, the solvent was evaporated and the reaction mixture was diluted with ethyl acetate. The organic layer was then washed with saturated NaHCO_3_, water, and brine. The combined organic layers were dried over MgSO_4_ and concentrated in vacuo to produce the crude product, which was purified via column chromatography (95:5 EtOAc:MeOH). The pure product **10a** was obtained as an off-white solid (0.041 g, 33%). TLC: EtOAc/MeOH 90:10 (Rf. 0.13). ^1^H NMR (400 MHz, CDCl_3_) δ 8.90 (s, 1H), 8.70 (d, *J* = 4.8 Hz, 1H), 8.40 (d, *J* = 4.8 Hz, 1H), 8.35 (s, 1H), 8.02 (d, *J* = 8.1 Hz, 1H), 7.43 (d, *J* = 8.0 Hz, 1H), 7.35 (dd, *J* = 4.8, 7.9 Hz, 1H), 7.30–7.13 (m, 10H), 6.85 (d, *J* = 7.7 Hz, 1H), 6.43 (t, *J* = 5.8 Hz, 1H (NH)), 4.47 (sext, *J* = 6.8 Hz, 1H), 3.47 (t, *J* = 7.7 Hz, 1H), 3.23 (m, 2H), 3.14 (quint, *J* = 6.6 Hz, 1H), 2.98 (m, 2H), 2.92 (dd, *J* = 6.9, 13.6 Hz, 1H), 2.85 (dd, *J* = 5.8, 13.5 Hz, 1H), 2.76 (dd, *J* = 6.4, 13.7 Hz, 1H), 2.47 (t, *J* = 7.7 Hz, 2H), and 1.68 (quint, *J* = 7.3 Hz, 2H). HRMS *m/z* calculated for C_32_H_34_N_4_O_2_Sna [M + Na]^+^: 561.2300. Found: 561.2272. The crude Boc-deprotected propyl–pyridyl thioether and nicotinuric acid (0.038 g, 0.21 mmol, 1 eq) produced **10b** as a light-yellow fluffy solid (0.062 g, 50%). TLC: EtOAc/MeOH 90:10 (Rf. 0.14). ^1^H NMR (400 MHz, CDCl_3_) δ 9.06 (s, 1H), 8.67 (d, *J* = 4.8 Hz, 1H), 8.36 (d, *J* = 4.8 Hz, 1H), 8.30 (s, 1H), 8.12 (t, *J* = 7.5 Hz, 2H), 7.41 (d, *J* = 7.8 Hz, 1H), 7.34 (dd, *J* = 4.9, 7.9 Hz, 1H), 7.25–7.06 (m, 8H), 6.96 (d, *J* = 9.2 Hz, 1H), 6.88 (t, *J* = 5.8 Hz, 1H), 4.27 (sext, *J* = 6.7 Hz, 1H), 4.03 (dd, *J* = 5.2, 16.2 Hz, 1H), 3.96 (dd, *J* = 5.4, 16.2 Hz, 1H), 3.55 (t, *J* = 7.3 Hz, 1H), 3.19 (m, 2H), 3.10 (quint, *J* = 6.5 Hz, 1H), 2.91 (dd, *J* = 6.8, 13.7 Hz, 1H), 2.83 (dd, *J* = 6.9, 16.6 Hz, 2H), 2.73 (dd, *J* = 5.6, 13.9 Hz, 1H), 2.65 (dd, *J* = 6.4, 13.6 Hz, 1H), 2.44 (t, *J* = 7.8 Hz, 2H), 1.66 (quint, *J* = 7.3 Hz, 2H), and 1.16 (t, *J =* 8.4 Hz, 1H (NH)). HRMS *m/z* calculated for C_34_H_38_N_5_O_3_S [M + H]^+^: 596.2695. Found: 596.2676.

*Protein Expression and Purification*—Codon-optimized full-length, Δ3-22 wild-type, and K421A/K424A CYP3A4 were produced as reported previously [17,36]. The full-length protein was used for assays and the truncated form was used for crystallization.

*Spectral Binding Titrations*—Equilibrium ligand binding to CYP3A4 was monitored using a Cary 300 spectrophotometer at 23 °C in 0.1 M phosphate buffer, pH 7.4, supplemented with 20% glycerol and 1 mM dithiothreitol. Inhibitors were dissolved in dimethyl sulfoxide (DMSO) and added to a 2 μM protein solution in small aliquots, with the final solvent concentration of <2%. The spectral dissociation constant (K_s_) was determined from quadratic fits to the titration plots.

*Thermal Denaturation*—Melting curves were recorded in 0.1 M phosphate buffer, pH 7.4, using a Cary 300 spectrophotometer. Protein (1 μM) was mixed with a ligand (20 μM) and incubated for 15 min at 23 °C. Protein denaturation was monitored at 260 nm using a 0.2 °C measurement step, 0.9 °C/min ramp rate, and 50–75 °C temperature range. The denaturation midpoint (melting temperature; T_m_) was determined from non-linear fittings to the melting curve, as described earlier [16].

*Inhibitory Potency Assays*—The inhibitory potency for the BFC *O*-debenzylase activity of CYP3A4 was evaluated fluorometrically in a soluble reconstituted system, and the IC_50_ values were derived from the [% activity] vs. [inhibitor] plots, as described in detail elsewhere [17].

*Kinetics of Ligand Binding*—The kinetics of ligand binding to CYP3A4 were measured at 427 nm in an SX.18MV stopped-flow apparatus (Applied Photophysics, UK), as previously described [16].

*H_2_O_2_-dependent heme depletion assay*—Heme bleaching in ligand-free and inhibitor-bound CYP3A4 (1.6 μM) was monitored at 23 °C in 0.1 M phosphate buffer, pH 7.4. After 10 min of preincubation of CYP3A4 with 16 μM inhibitors, heme decay was initiated by the addition of 10 mM H_2_O_2_ (final concentration) and monitored at 420 nm for 2 h. The percentage of heme destroyed was calculated relative to that in ligand-free CYP3A4, considered as 100% decay.

*Crystallization of the inhibitory complexes*—**5a**,**b** and **8a**–**c** were co-crystallized with the wild-type Δ3-22 CYP3A4 and **10a**,**b** with the K421A/K424A mutant. Crystals were grown using a microbatch method under paraffin oil. Prior to crystal setup, CYP3A4 (60–70 mg/mL in 20–100 mM phosphate, pH 7.4) was incubated with a 2-fold ligand excess and centrifuged to remove the precipitate. The supernatant containing the inhibitor-bound CYP3A4 (0.4–0.6 μL) was mixed with 0.4–0.6 μL of crystallization solution, containing 8–12% polyethylene glycol 3350 and either 80 mM taximate, pH 6.0–7.0 (Hampton Research; for **5a**, **8a** and **10a**), 60 mM malate, pH 7.0 (for **5b** and **8b**), or 70 mM succinate, pH 7.0 (for **8c** and **10b**). Crystals were grown at room temperature for 2–3 days and cryoprotected with Paratone-N before freezing in liquid nitrogen.

*Determination of the X-ray Structures*—X-ray diffraction data were collected at the Stanford Synchrotron Radiation Lightsource beamlines 9–2 and 12-2, and the Advanced Light Source beamline 8.2.1. The crystal structures were solved by molecular replacement with PHASER [43]. The 5VCC structure was used as a search model for the **5a**,**b** and **8a**–**c** bound complexes crystallized in the I222 space group. For the **10a**- and **10b**-bound CYP3A4, which produced crystals in the C2 space group, the search model was molecule A of the 7KVH structure. Ligands were built with eLBOW [44] and manually fitted to the density with COOT [45]. The initial models were rebuilt and refined with COOT and PHENIX [44]. Polder omit electron density maps and local correlation coefficients (listed in Table S3) were calculated with PHENIX. Data collection and refinement statistics are summarized in Tables S1 and S2. The atomic coordinates and structural factors for the **5a**-, **5b**-, **8a**-, **8b**-, **8c**-, **10a**-, and **10b**-bound CYP3A4 were deposited in the Protein Data Bank with the ID codes 7UF9, 7UFA, 7UFB, 7UFC, 7UFD, 7UFE, and 7UFF, respectively.

## Figures and Tables

**Table 1:** Properties of series-VI inhibitors.

Compound	λ_max_ (nm)	A_421/417_ ^a^	ΔA_max_ ^b^	K_s_ ^c^	IC_50_ ^d^	IC_50_/K_s_	ΔT_m_ ^e^
	Ferric/Ferrous		%	μM	μM		°C
*R_1_-meta-N-pyridine; R_3_-Boc*							
*Pyridyl–peptidyl linker*							
**4** (*R*, *S*)	421/- ^f^	0.99	98	1.36 ± 0.12	1.6 ± 0.2	1.2	1.9
*R_1_-para-N-pyridine; R_3_-Boc*							
*Pyridyl–ethyl linker*							
**5a** (*R*, *S*)	422/443	1.07	105	0.034 ± 0.005	0.31 ± 0.03	9.1	5.2
*Pyridyl–propyl linker*							
**5b** (*R*, *S*)	423/443	1.06	126	0.014 ± 0.002	0.16 ± 0.02	11.4	5.0
*R_1_/R_3_-meta-N-pyridine*							
*Pyridyl–methyl linker*							
**8a** (*R*, *S*)	421/443	1.03	96	0.120 ± 0.010	0.60 ± 0.04	5.0	3.6
*Pyridyl–ethyl linker*							
**8b** (*R*, *S*)	421/443	1.03	109	0.053 ± 0.009	0.82 ± 0.09	15.5	4.2
**8c** (*S*, *S*)	422/443	1.08	103	0.015 ± 0.002	0.21 ± 0.03	14.0	4.5
*R_1_-meta-N-pyridine; pyridyl–propyl linker*							
*R_3_-meta-N-pyridine; single amide bond linker*							
**10a** (*R*, *S*)	422/444	1.11	111	0.008 ± 0.001	0.15 ± 0.02	18.8	7.2
*R_3_-meta-N-pyridine; double amide bond linker*							
**10b** (*R*, *S*)	422/443	1.10	110	0.009 ± 0.001	0.21 ± 0.02	23.3	7.3

**^a^**—Ratio between the absorption maxima of ferric ligand-bound and ligand-free CYP3A4. **^b^**—Maximal absorbance change (peak-to-trough in the difference spectrum) relative to that induced by ritonavir. **^c^**—Spectral dissociation constants for the inhibitory complexes derived from the titration plots (right insets in Figure 3A–C). **^d^**—Inhibitory potency for the BFC debenzylase activity measured in a soluble reconstituted system with cytochrome P450 reductase. **^e^**—Ligand-dependent change in the melting temperature of CYP3A4. **^f^**—Ferrous CYP3A4-**4** complex does not absorb at 442–443 nm.

**Table 2:** Parameters for the ligand-binding and heme-bleaching kinetics.

	*k*_fast_ ^a^	*k*_H2O2_ ^b^	Heme Destroyed ^c^
Compound	s^−1^	10^3^ × min^−1^	(%)
**4** (R, S)	13.8 ± 1.5 (27%) ^d^	0.70 ± 0.08	44 ± 5
**5a** (R, S)	22.5 ± 3.1 (34%)	0.31 ± 0.04	22 ± 3
**5b** (R, S)	18.2 ± 1.8 (31%)	0.31 ± 0.03	22 ± 2
**8a** (R, S)	10.3 ± 1.2 (26%)	0.66 ± 0.10	39 ± 4
**8b** (R, S)	5.6 ± 0.6(24%)	0.44 ± 0.06	32 ± 3
**8c** (S, S)	15.0 ± 2.1 (28%)	0.32 ± 0.03	24 ± 2
**10a** (R, S)	15.4 ± 1.8 (30%)	0.17 ± 0.02	13 ± 2
**10b** (R, S)	13.7 ± 1.5 (29%)	0.24 ± 0.03	19 ± 3
*Analogues from previous series*			
**4b** (R, S)	13.2 ± 1.8 (31%)	0.64 ± 0.07	39 ± 5
**4f** (R, S)	11.4 ± 1.4 (32%)	0.60 ± 0.08	37 ± 4
**4h** (S, S)	13.8 ± 2.0 (28%)	0.32 ± 0.03	22 ± 3
**3a** (R, S)	12.5 ± 1.3(36%)	0.21 ± 0.03	18 ± 2
Ritonavir	7.5 ± 1.0 (27%)	0.35 ± 0.05	26 ± 3
Ligand-free		4.2 ± 0.06	100

**^a^**—Rate constant for the fast kinetic phase of the ligand-binding reaction. **^b, c^**—Rate constant for the H_2_O_2_-dependent heme-bleaching reaction and percentage of heme destroyed, respectively. **^d^**—Percentage of the absorbance change taking place during the fast kinetic phase of the ligand-binding reaction. Data represent the mean of triplicate measurements ± standard deviation.

**Table 3:** Structural features of the inhibitory complexes.

Compound	5a	5b	8a	8b	8c	10a	10b
	(*R*, *S*)	(*R*, *S*)	(*R*, *S*)	(*R*, *S*)	(*R*, *S*)	(*R*, *S*)	(*R*, *S*)
Fe–N bond							
distance (Å)	2.06	2.08	2.29	2.10	2.04	2.16	2.21
angle (°) ^a^	5	7	0	5	3	2	0
Pyridine ring	20	25	25	15	20	40	40
rotation (°) ^b^							
I-helix dis-							
placement (Å) ^c^	1.25–1.60	1.41–1.52	0.63–1.17	0.39–0.62	0.40–1.19	2.12–2.15	2.12–2.16
H-bond (Å)							
to Ser119 ^d^	2.96	2.55	-	-	2.61	2.76	2.46
to Thr224 ^e^	-	-	3.29	-	2.93	-	-
Pyridine–R_2_ ring angle; overlap	5°; Half	75°; Full	25°; Full	52°; Half	35°; Half	70°; Half	55°; Half
Phe304–R_1_ ring angle; overlap	25°; Partial	55°; Half	53°; Partial	90°; None	100°; Partial	50°; Half	55°; Half
S–π interaction ^f^	^−^	^−^	^−^	^−^	^−^	+	+
End-group	106–108	108,215	106–108	57, 76	57, 76	57, 106–108	53, 57, 76
contacts	374	482	215, 374	106–108, 215	107, 108	213, 224, 374	106–108
				224	215, 374		213,215
							224, 372, 374

**^a^**—Angle between the heme and heme-ligating pyridine. **^b^**—Angle between the planes passing through the heme-ligating pyridine and the NB-ND heme atoms. **^c^**—Distance between C_α_ atoms of Phe304 and Ala305 in the inhibitor-bound and ligand-free CYP3A4 (5VCC structure). **^d^**—H-bond between the inhibitor’s carbonyl oxygen atom and Ser119 hydroxyl group. **^e^**—H-bond between the inhibitor’s end-pyridine N atom and Thr224 hydroxyl group. **^f^**—Interaction between the backbone sulfur atom and Phe108 ring.

## Data Availability

Structure factors and coordinate files for the described structures are publicly available in the Protein Data Base (https://www.rcsb.org/).

## Supplementary Materials

The following supporting information can be downloaded at: https://www.mdpi.com/article/10.3390/ijms23137291/s1.

## Abbreviations

CYP3A4	Cytochrome P450 3A4
BFC	7-benzyloxy-4-(trifluoromethyl)coumarin
SAR	Structure–activity relationship
