# Supplementary material for: Interaction of CYP3A4 with Rationally Designed Ritonavir Analogues: Impact of Steric Constraints Imposed on the Heme-Ligating Group and the End-Pyridine Attachment
Source: Int J Mol Sci. 2022 Jun 30;23(13):7291. doi: 10.3390/ijms23137291 (PMC9266530; doi:10.3390/ijms23137291)
Supplement: Supplementary file 1 [file ijms-23-07291-s001.zip › ijms-1776756-supplementary.pdf]

Interaction of human drug-metabolizing CYP3A4 with rationally designed ritonavir analogues: Impact of steric constraints imposed on the heme-ligating group and the end-pyridine attachment

Eric R. Samuels and Irina F. Sevrioukova

SUPPLEMENTARY MATERIALS

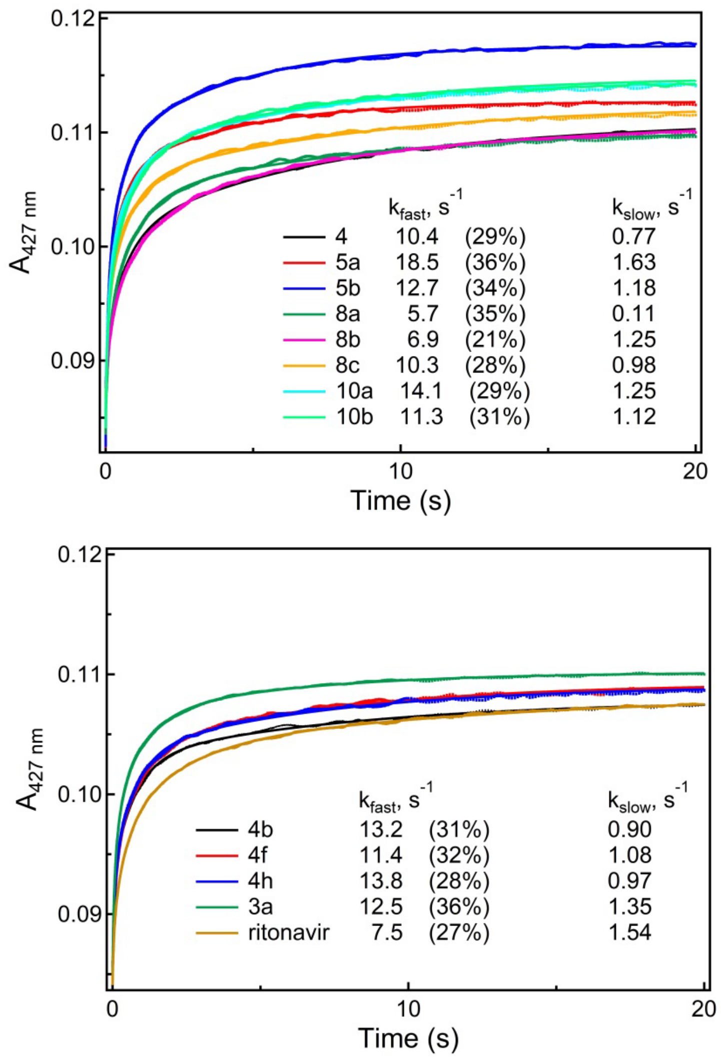

Figure S1. Kinetics of ligand binding to CYP3A4. Kinetic traces were recorded by mixing 2 μM CYP3A4 with 20 μM solutions of series VI analogues (upper panel) or previously reported compounds and ritonavir (lower panel). Within the studied time interval, all reactions were biphasic. Fittings are shown as solid lines. The derived rate constants for the fast and slow phases and the percentage of the fast phase are indicated.

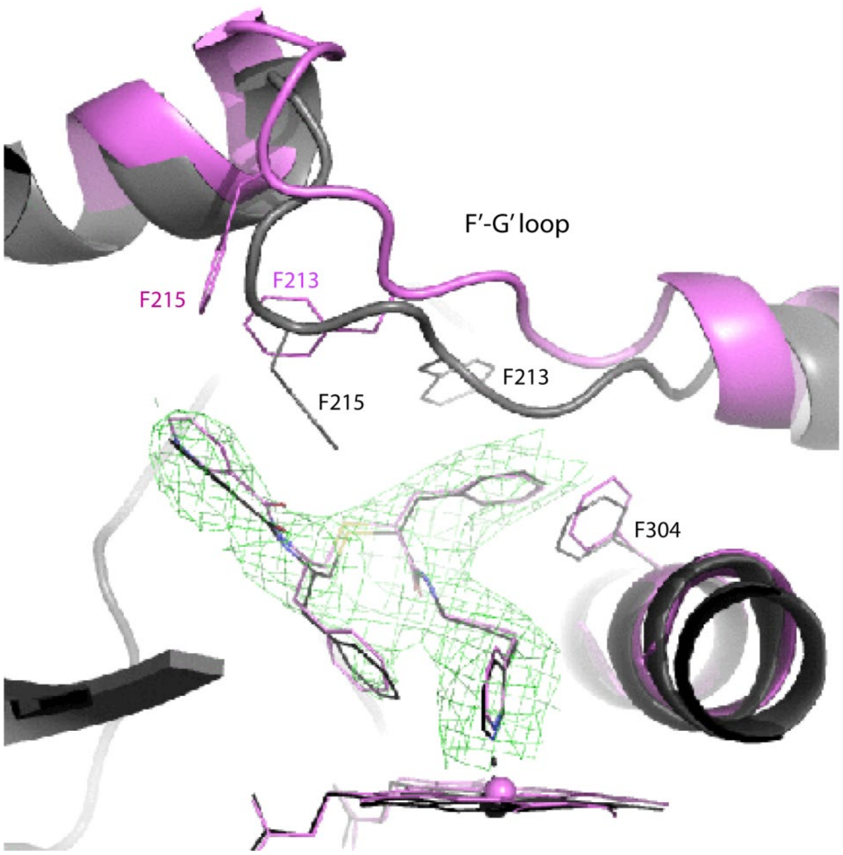

Figure S2. Superposition of **10a**–bound CYP3A4 crystallized in the I222 (magenta) and C2 space group (gray; molecule A). The ligand binding mode is only slightly affected by differences in crystal packing. Green mesh is polder omit electron density map contoured at 3 σ level.

Table S1. Data collection and refinement statistics

Ligand	**5a**	**5b**	**8a**	**8b**
CYP3A4	WT	WT	WT	WT
PDB ID	7UF9	7UFA	7UFB	7UFC
Data statistics				
Space group	I222	I222	I222	I222
Unit cell	*a, b, c* = 77 x 101 x 128 Å; α, β, γ = 90°	*a, b, c* = 77 x 102 x 126 Å; α, β, γ = 90°	*a, b, c* = 77 x 102 x 128 Å; α, β, γ = 90°	*a, b, c* = 78 x 103 x 128 Å; α, β, γ = 90°
Resolution range (Å)	79.53-2.45 (2.58-2.45) [a]	50.86-2.50 (2.64-2.50)	79.62-2.25 (2.37-2.25)	80.35-2.35 (2.48-2.35)
Total reflections	82,809 (12,979)	124,746 (17,945)	186,616 (26,677)	82,093 (12,824)
Unique reflections	18,319 (2,694)	17,305 (2,503)	24,033 (3,473)	20,611 (3,070)
Redundancy	4.5 (4.6)	7.2 (7.2)	7.8 (7.7)	4.0 (4.2)
Completeness	98.1 (99.5)	99.8 (100.0)	99.5 (99.6)	95.3 (98.0)
Average $I/\sigma I$	12.5 (1.1)	9.8 (1.0)	13.8 (1.3)	9.2 (1.1)
R_{merge}	0.047 (1.630)	0.089 (2.524)	0.059 (1.604)	0.066 (2.695)
R_{pim}	0.024 (0.842)	0.036 (1.006)	0.022 (0.610)	0.036 (1.362)
CC ½	0.999 (0.416)	0.998 (0.403)	0.999 (0.395)	0.994 (0.480)
Refinement statistics				
R/R_{free} [b]	22.5/25.5	24.7/28.4	21.0/25.8	20.8/25.4
Number of atoms:				
Protein	3645	3735	3350	3727
Solvent	0	6	20	7
R.m.s. deviations				
Bond lengths, Å	0.004	0.003	0.003	0.003
Bond angles, °	0.634	0.558	0.517	0.535
Wilson B-factor, $Å^2$	85	79	70	77
Average *B*-factor, $Å^2$:				
Protein	119	119	101	107
Ligand	137	119	102	107
Ramachandran plot [d] (residues; %)				
Preferred	413 (94.3%)	421 (94.6%)	445 (96.7%)	434 (96.2%)
Allowed	23 (5.2%)	23 (5.2%)	15 (3.3%)	27 (3.8%)
Outliers	2 (0.5%)	1 (0.2%)	none	none

[a]Values in brackets are for the highest resolution shell.
[b]R_{free} was calculated from a subset of 5% of the data that were excluded during refinement.
[c]Values for two molecules in the asymmetric unit.
[d]Analyzed with PROCHECK.

Table S2. Data collection and refinement statistics

Ligand	**8c**	**10a**	**10b**
CYP3A4	WT	K421A/K424A	K421A/K424A
PDB ID	7UFD	7UFE	7UFF
Data statistics			
Space group	I222	C2	C2
Unit cell	*a, b, c* = 78 x103 x 130 Å; α, β, γ = 90	*a, b, c* = 154 x 97 x 94 Å; α, β, γ = 90 x 124 x 90°	*a, b, c* = 157 x 98 x 95 Å; α, β, γ = 90 x 125 x 90°
Resolution range (Å)	80.52-2.90 (3.06-2.90)[a]	77.63-2.40 (2.53-2.40)	77.75-2.70 (2.85-2.70)
Total reflections	57,848 (8,841)	148,105 (22,323)	183,071 (22,905)
Unique reflections	11,818 (1,714)	43,861 (6,382)	30,683 (4,113)
Redundancy	4.9 (5.2)	3.4 (3.5)	6.0 (5.5)
Completeness	99.8 (99.9)	98.7 (98.9)	95.1 (88.6)
Average $I/\sigma I$	6.3 (2.5)	8.3 (1.1)	8.5 (1.0)
R_{merge}	0.174 (1.203)	0.063 (1.257)	0.115 (1.774)
R_{pim}	0.089 (0.570)	0.039 (0.777)	0.050 (0.818)
CC ½	0.972 (0.354)	0.998 (0.506)	0.997 (0.405)
Refinement statistics			
R/R_{free}[b]	20.1/27.8	22.1/25.3	24.4/28.1
Number of atoms:			
Protein	3727	3706/3575[c]	3685/3575[c]
Solvent	0	53	13
R.m.s. deviations0.003	0.002	0.002	0.002
Bond angles, °	0.476	0.503	0.511
Wilson B-factor, Å^2	94	71	83
Average *B*-factor, Å^2:			
Protein	107	90/111[c]	92/123[c]
Ligand	103	80/90[c]	83/115[c]
Ramachandran plot[d] (residues; %)			
Preferred	429 (95.1%)	820 (92.3%)	822 (93.3%)
Allowed	22 (4.9%)	67 (7.6%)	58 (6.6%)
Outliers	none	1 (0.1%)	1 (0.1%)

[a]Values in brackets are for the highest resolution shell.
[b]R_{free} was calculated from a subset of 5% of the data that were excluded during refinement.
[c]Values for two molecules in the asymmetric unit.
[d]Analyzed with PROCHECK.

Table S3. Local correlation coefficients (CC) and CC_{peak} between three polder maps (F_{obs} = $|F_{model}|$)

Map 1 (m1), calculated F_{obs} assuming that the omitted ligand atoms are present. Map 2 (m2), calculated F_{obs} assuming that the omitted ligand atoms are not present. Map 3 (m3), polder map calculated using the experimental data.

Ligand/PDB ID		m1-m2		m1-m3		m2-m3	
		CC	CC_{peak}	CC	CC_{peak}	CC	CC_{peak}
**5a**	7UF9	0.6992	0.6672	0.9230	0.8715	0.6336	0.5914
**5b**	7UFA	0.4962	0.4570	0.8256	0.7698	0.4460	0.4101
**8a**	7UFB	0.7025	0.7522	0.9062	0.8803	0.7123	0.7491
**8b**	7UFC	0.5861	0.5493	0.8972	0.8544	0.5939	0.5425
**8c**	7UFD	0.6629	0.6702	0.9049	0.8783	0.5949	0.6046
**10a**	7UFE	0.5353	0.5100	0.9200	0.8496	0.5071	0.4826
**10b**	7UFF	0.5257	0.5584	0.8700	0.8496	0.4717	0.4749

Correlation coefficients for polder maps were calculated with PHENIX.

For all ligands, pairwise comparisons of correlation coefficients indicate that m3 correlates best with m1. This means that the polder maps m3 (displayed in Figures 3, 5 and 7 of the main text) show the omitted ligand atoms.*

*Liebschner D., Afonine P.V.; Moriarty N.W.; Poon B.K.; Sobolev O.V.; Terwilliger T.C.; Adams P.D. Polder maps: improving OMIT maps by excluding bulk solvent. *Struct. Biol.* **2016**, D73, 148-157.

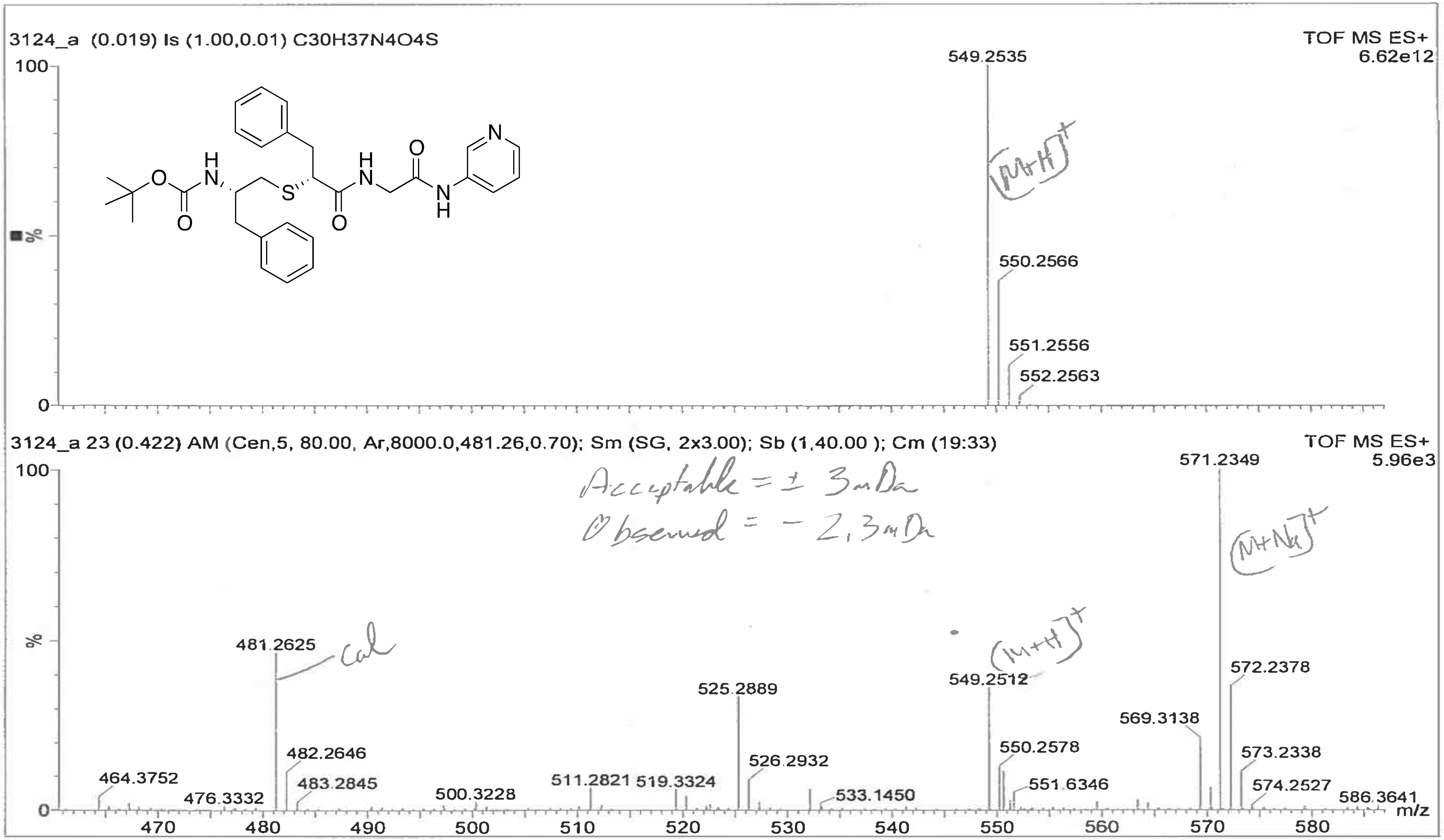
3124_a (0.019) Is (1.00,0.01) C30H37N4O4S
TOF MS ES+
6.62e12
549.2535
550.2566
551.2556
552.2563
[M+H]+
3124_a 23 (0.422) AM (Cen,5, 80.00, Ar,8000.0,481.26,0.70); Sm (SG, 2x3.00); Sb (1,40.00); Cm (19:33)
TOF MS ES+
5.96e3
Acceptable = ± 3mDa
Observed = − 2.3mDa
571.2349
[M+Na]+
481.2625
cal
[M+H]+
549.2512
572.2378
525.2889
569.3138
550.2578
573.2338
482.2646
526.2932
464.3752
483.2845
511.2821
519.3324
551.6346
574.2527
476.3332
500.3228
533.1450
586.3641
m/z
%

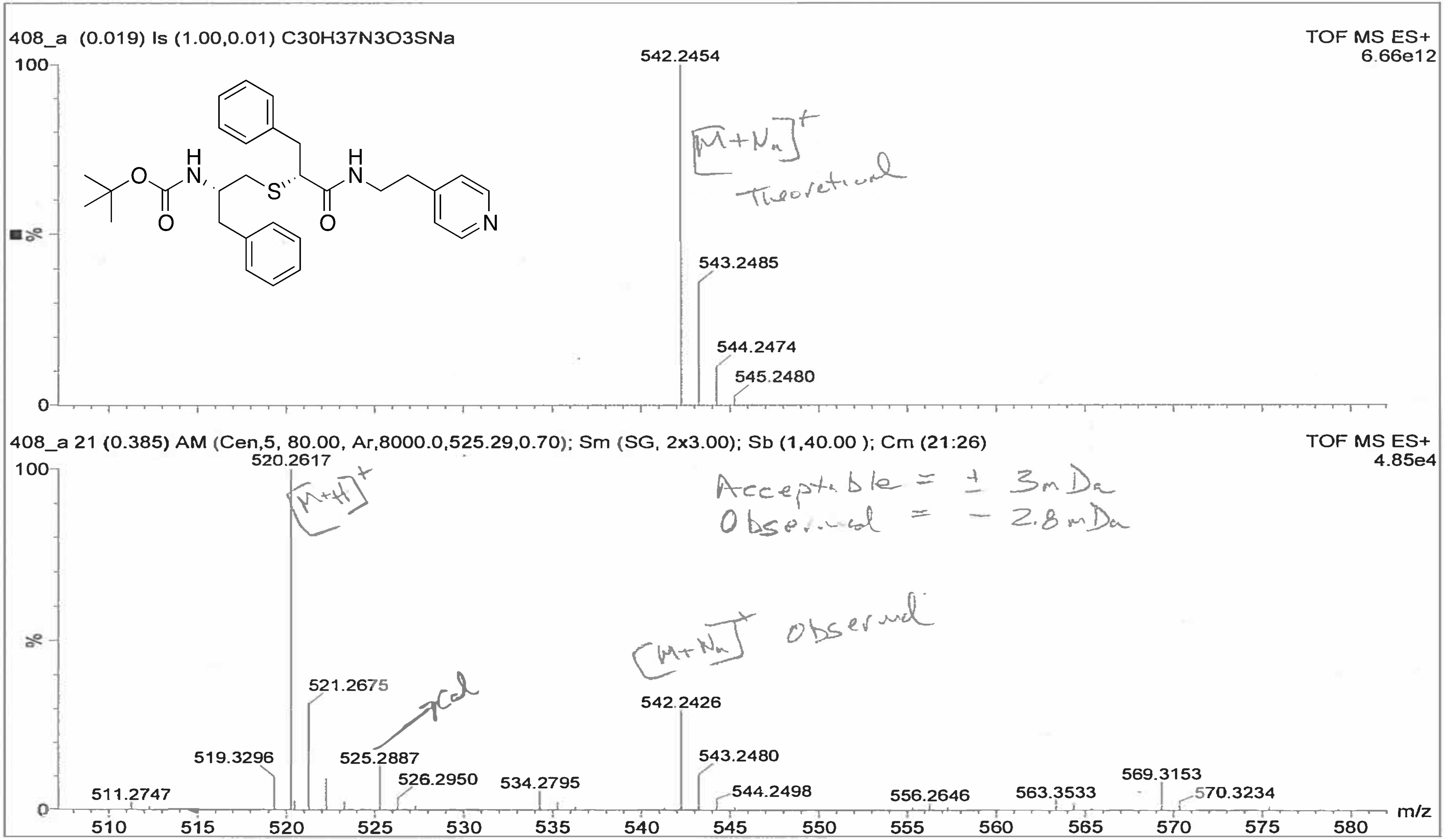
408_a (0.019) Is (1.00,0.01) C30H37N3O3SNa
TOF MS ES+
6.66e12
542.2454
[M+Na]+
Theoretical
543.2485
544.2474
545.2480
408_a 21 (0.385) AM (Cen,5, 80.00, Ar,8000.0,525.29,0.70); Sm (SG, 2x3.00); Sb (1,40.00); Cm (21:26)
TOF MS ES+
4.85e4
520.2617
[M+H]+
Acceptable = ± 3mDa
Observed = − 2.8mDa
[M+Na]+ observed
521.2675
542.2426
519.3296
525.2887
543.2480
526.2950
534.2795
569.3153
511.2747
544.2498
556.2646
563.3533
570.3234
m/z
%
100
0

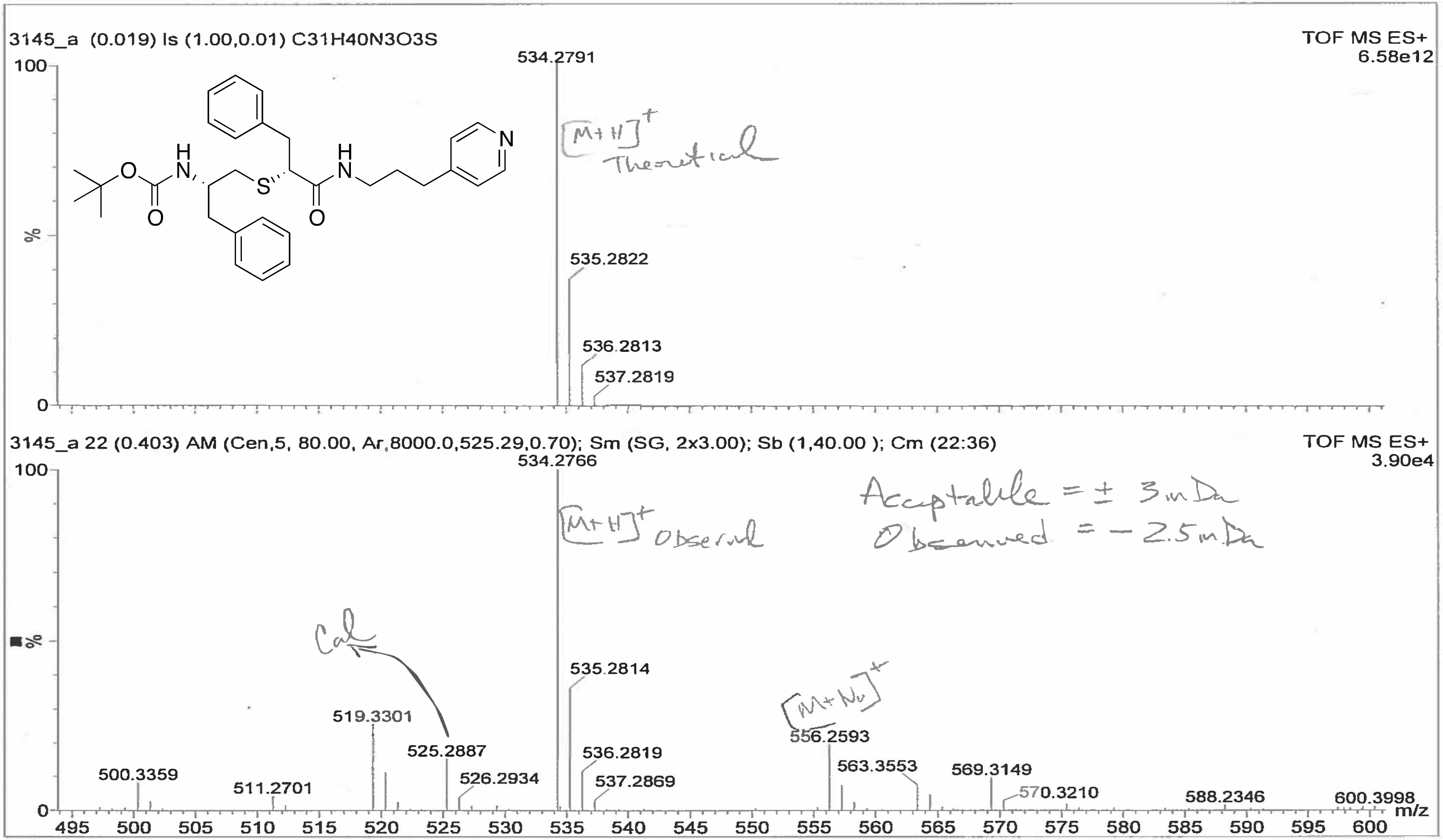
3145_a (0.019) Is (1.00,0.01) C31H40N3O3S
TOF MS ES+
6.58e12
534.2791
[M+H]+ Theoretical
535.2822
536.2813
537.2819
100
%
0
3145_a 22 (0.403) AM (Cen,5, 80.00, Ar,8000.0,525.29,0.70); Sm (SG, 2x3.00); Sb (1,40.00); Cm (22:36)
TOF MS ES+
3.90e4
534.2766
[M+H]+ Observed
Acceptable = ± 3 mDa
Observed = − 2.5 mDa
Cal
535.2814
[M+Na]+
519.3301
525.2887
556.2593
536.2819
563.3553
569.3149
500.3359
526.2934
511.2701
537.2869
570.3210
588.2346
600.3998
m/z
495 500 505 510 515 520 525 530 535 540 545 550 555 560 565 570 575 580 585 590 595 600

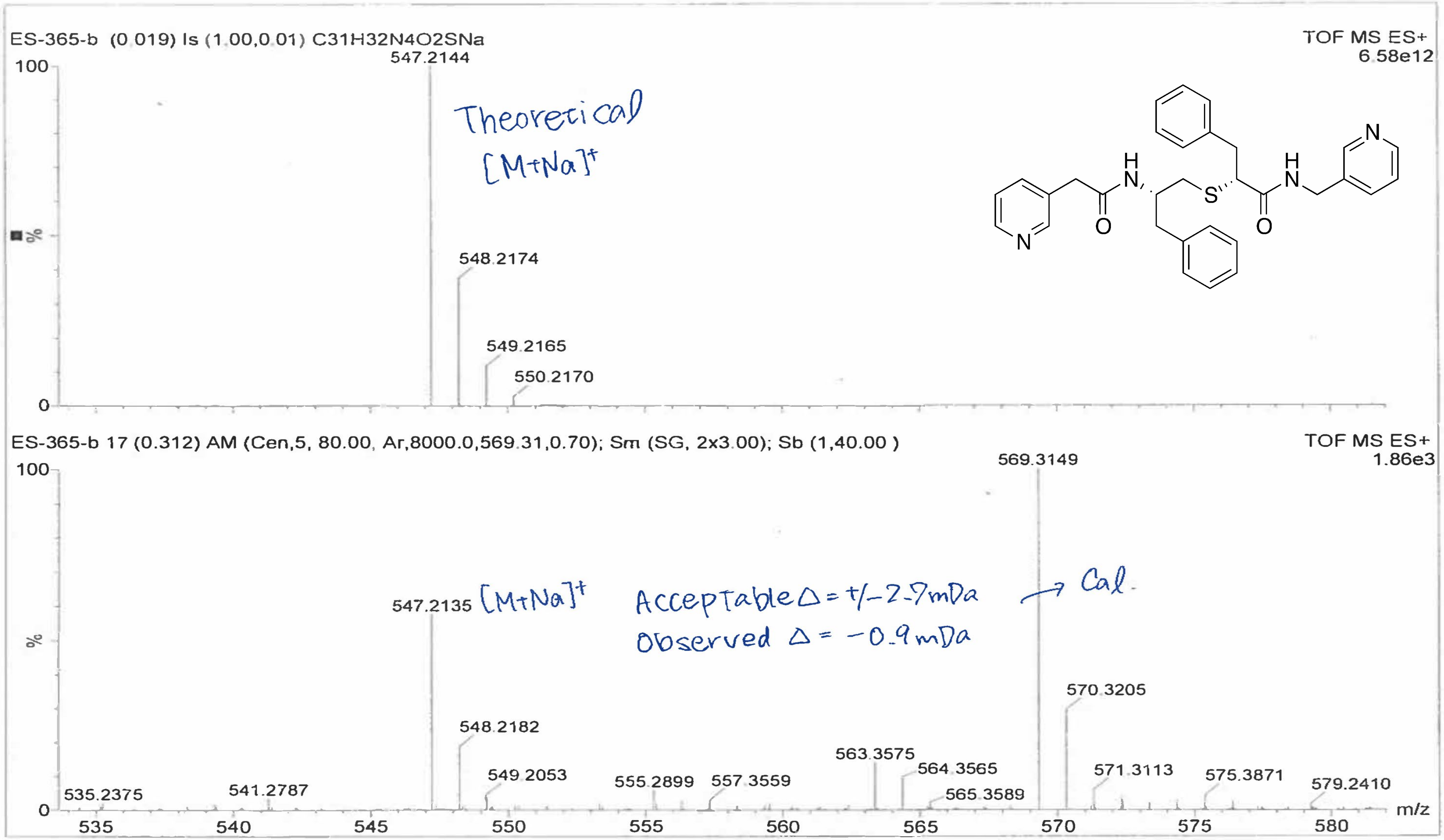
ES-365-b (0.019) Is (1.00,0.01) C31H32N4O2SNa
TOF MS ES+
6.58e12
547.2144
548.2174
549.2165
550.2170
100
%
0
Theoretical
[M+Na]+
N
H
N
S
H
N
O
O
N
ES-365-b 17 (0.312) AM (Cen,5, 80.00, Ar,8000.0,569.31,0.70); Sm (SG, 2x3.00); Sb (1,40.00)
TOF MS ES+
1.86e3
569.3149
547.2135 [M+Na]+
Acceptable Δ = +/-2.7mDa
Observed Δ = -0.9mDa
→ Cal.
570.3205
548.2182
563.3575
564.3565
549.2053
555.2899
557.3559
565.3589
571.3113
575.3871
579.2410
535.2375
541.2787
535
540
545
550
555
560
565
570
575
580
m/z

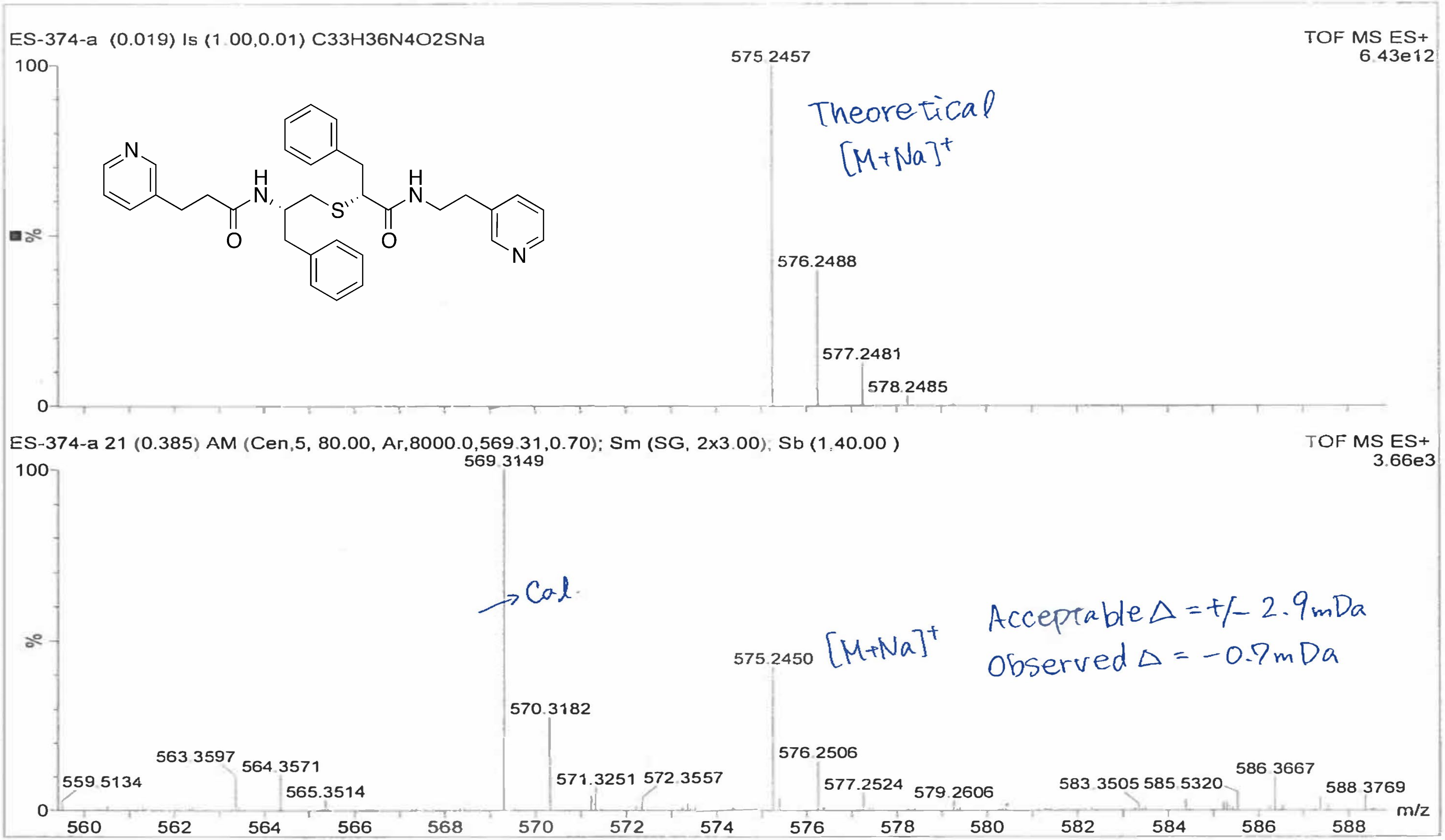
ES-374-a (0.019) Is (1.00,0.01) C33H36N4O2SNa
TOF MS ES+
6.43e12
575.2457
576.2488
577.2481
578.2485
Theoretical [M+Na]+
ES-374-a 21 (0.385) AM (Cen,5, 80.00, Ar,8000.0,569.31,0.70); Sm (SG, 2x3.00); Sb (1,40.00)
TOF MS ES+
3.66e3
569.3149
Cal.
570.3182
575.2450
[M+Na]+
Acceptable Δ = +/- 2.9mDa
Observed Δ = -0.7mDa
559.5134
563.3597
564.3571
565.3514
571.3251
572.3557
576.2506
577.2524
579.2606
583.3505
585.5320
586.3667
588.3769
m/z
%
100
0

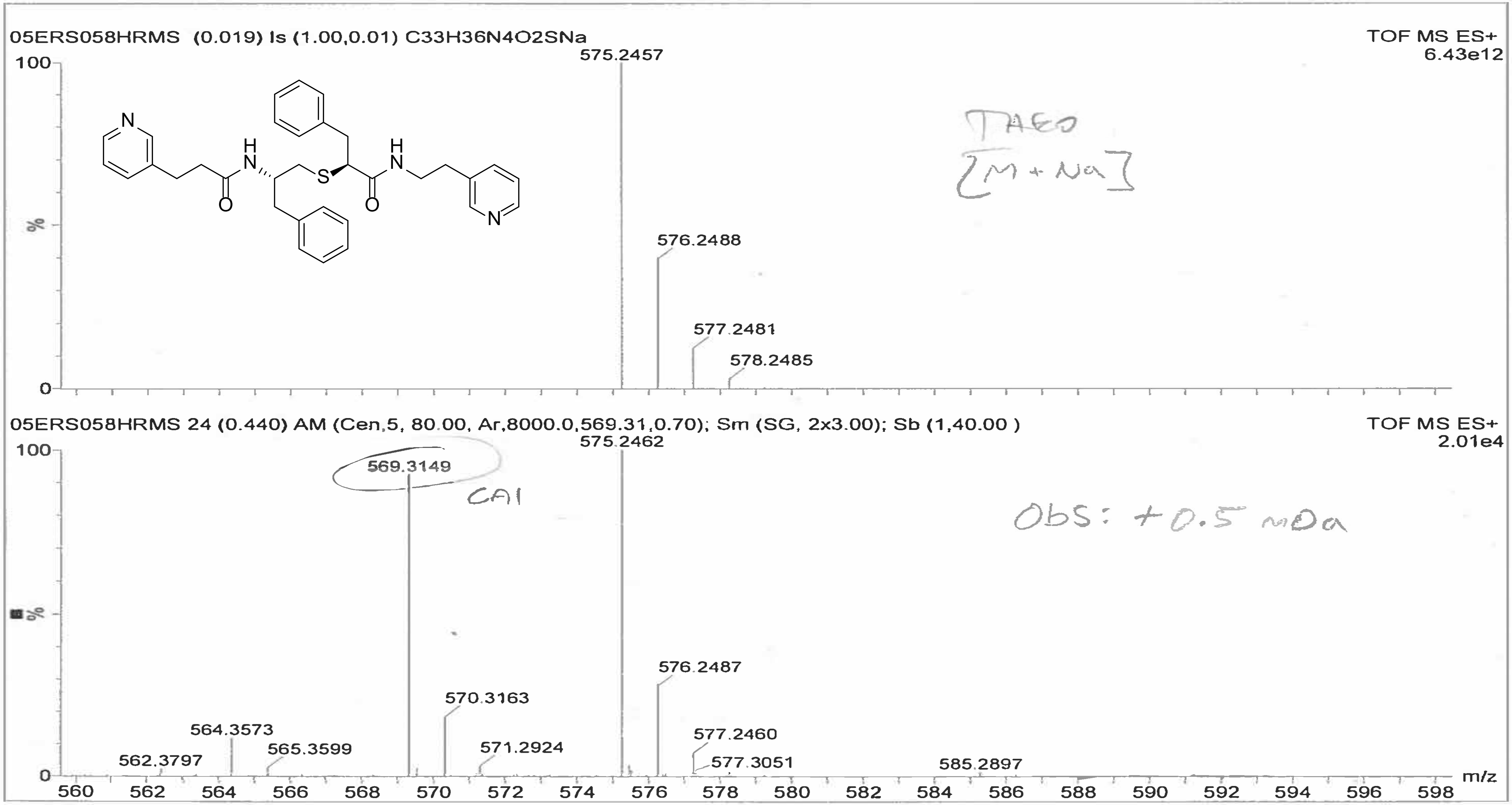
05ERS058HRMS (0.019) Is (1.00,0.01) C33H36N4O2SNa
TOF MS ES+
6.43e12
575.2457
576.2488
577.2481
578.2485
TAEO
[M + Na]
05ERS058HRMS 24 (0.440) AM (Cen,5, 80.00, Ar,8000.0,569.31,0.70); Sm (SG, 2x3.00); Sb (1,40.00)
TOF MS ES+
2.01e4
575.2462
569.3149
CAl
Obs: +0.5 mDa
576.2487
570.3163
564.3573
565.3599
562.3797
571.2924
577.2460
577.3051
585.2897
m/z

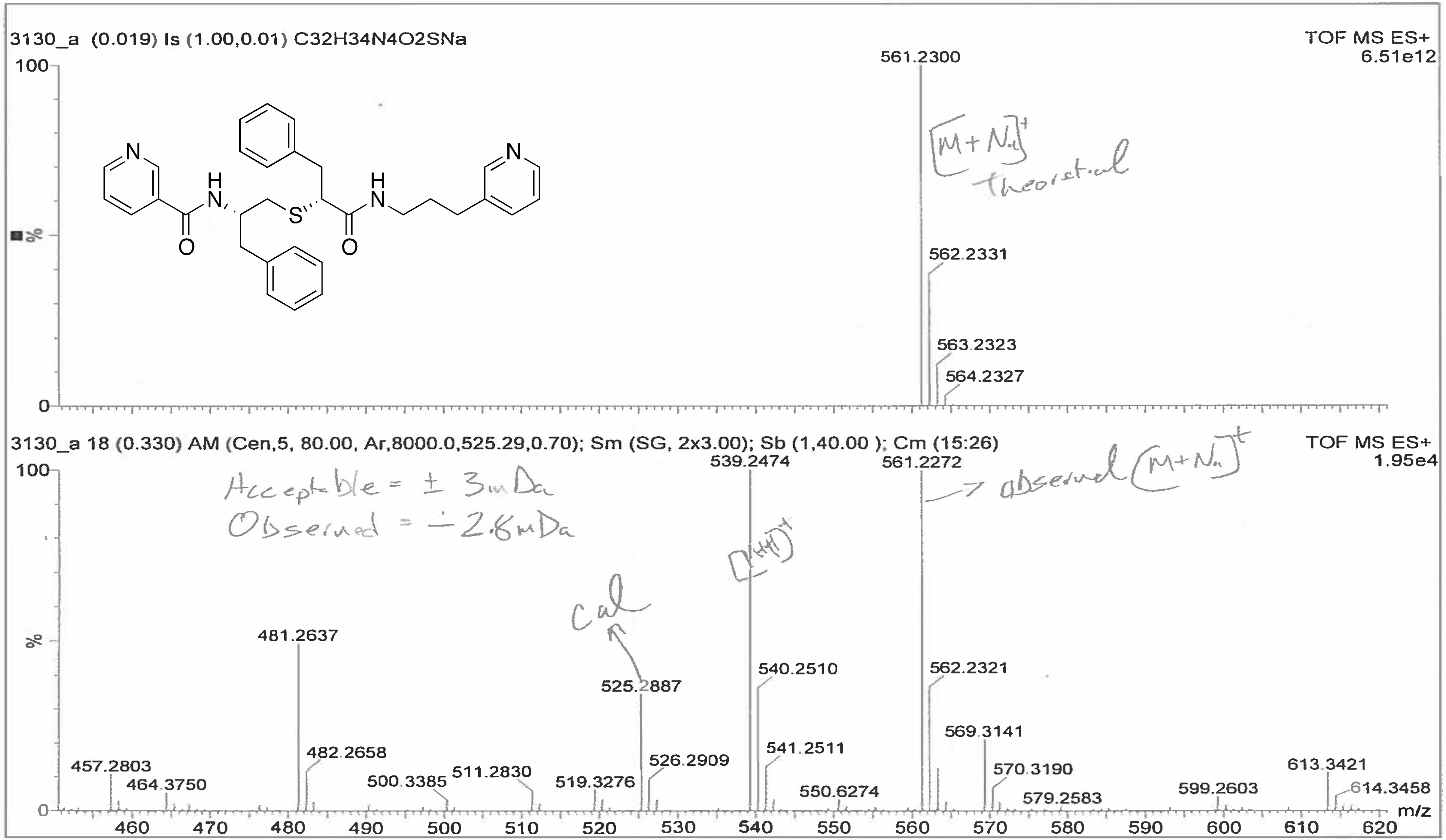
3130_a (0.019) Is (1.00,0.01) C32H34N4O2SNa
TOF MS ES+
6.51e12
561.2300
[M+Na]+
Theoretical
562.2331
563.2323
564.2327
3130_a 18 (0.330) AM (Cen,5, 80.00, Ar,8000.0,525.29,0.70); Sm (SG, 2x3.00); Sb (1,40.00); Cm (15:26)
TOF MS ES+
1.95e4
539.2474
561.2272
observed [M+Na]+
Acceptable = ± 3mDa
Observed = -2.8mDa
[M+H]+
Cal
481.2637
525.2887
540.2510
562.2321
569.3141
457.2803
464.3750
482.2658
500.3385
511.2830
519.3276
526.2909
541.2511
550.6274
570.3190
579.2583
599.2603
613.3421
614.3458
m/z
%
100
0

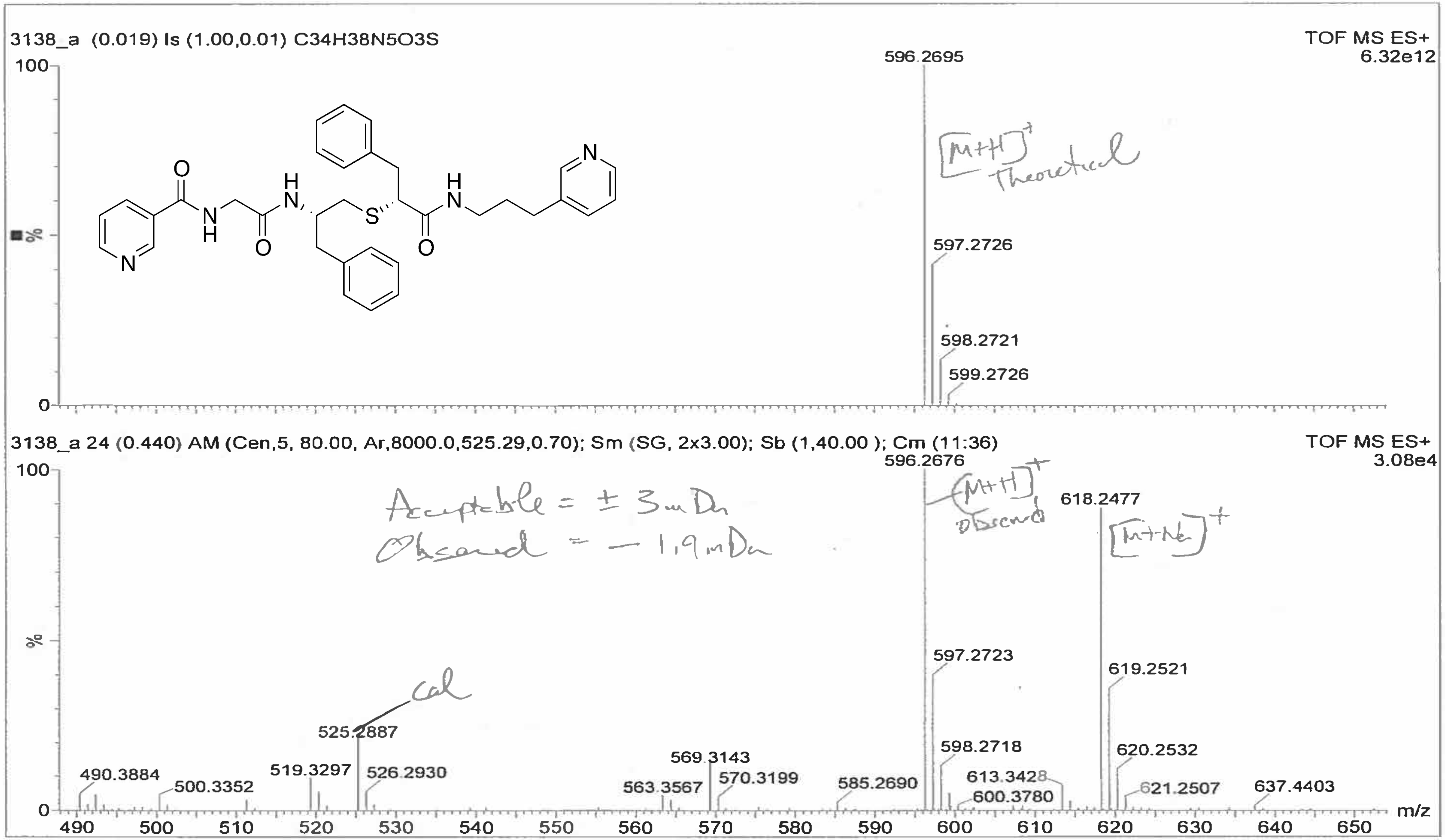
3138_a (0.019) Is (1.00,0.01) C34H38N5O3S
TOF MS ES+
6.32e12
596.2695
[M+H]+ Theoretical
597.2726
598.2721
599.2726
3138_a 24 (0.440) AM (Cen,5, 80.00, Ar,8000.0,525.29,0.70); Sm (SG, 2x3.00); Sb (1,40.00); Cm (11:36)
TOF MS ES+
3.08e4
596.2676
[M+H]+ Observed
618.2477
[M+Na]+
Acceptable = ± 3mDa
Observed = − 1.9mDa
597.2723
619.2521
Cal
525.2887
598.2718
620.2532
569.3143
490.3884
519.3297
526.2930
613.3428
500.3352
563.3567
570.3199
585.2690
600.3780
621.2507
637.4403
m/z

1H spectrum

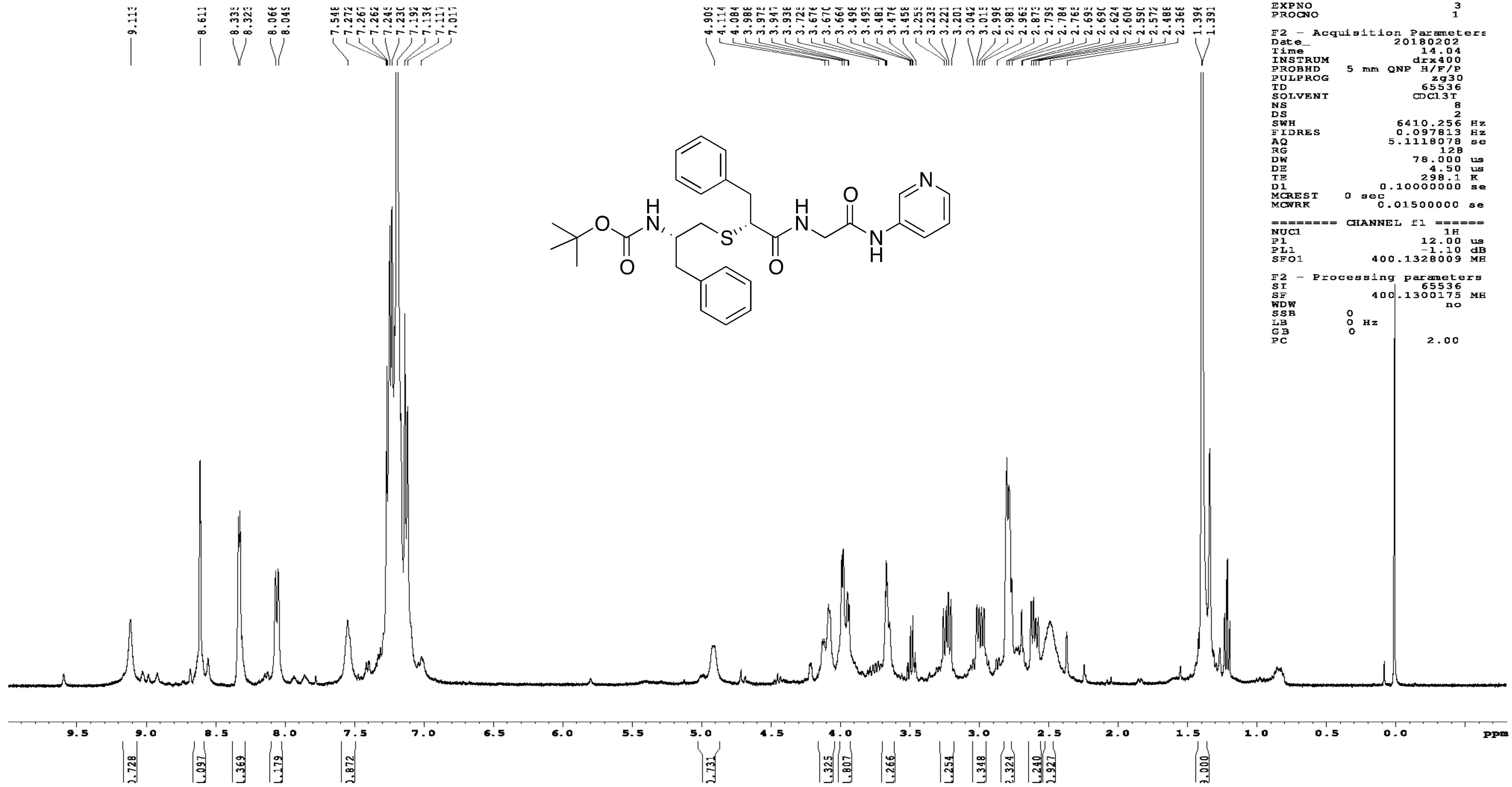

Current Data Parameters
NAME 03ERS124
EXPNO 3
PROCNO 1

F2 - Acquisition Parameters
Date_ 20180202
Time 14.04
INSTRUM drx400
PROBHD 5 mm QNP H/F/P
PULPROG zg30
TD 65536
SOLVENT CDCl3T
NS 8
DS 2
SWH 6410.256 Hz
FIDRES 0.097813 Hz
AQ 5.1118078 se
RG 128
DW 78.000 us
DE 4.50 us
TE 298.1 K
D1 0.10000000 se
MCREST 0 sec
MCWRK 0.01500000 se

======== CHANNEL f1 ======
NUC1 1H
P1 12.00 us
PL1 -1.10 dB
SFO1 400.1328009 MH

F2 - Processing parameters
SI 65536
SF 400.1300175 MH
WDW no
SSB 0
LB 0 Hz
GB 0
PC 2.00

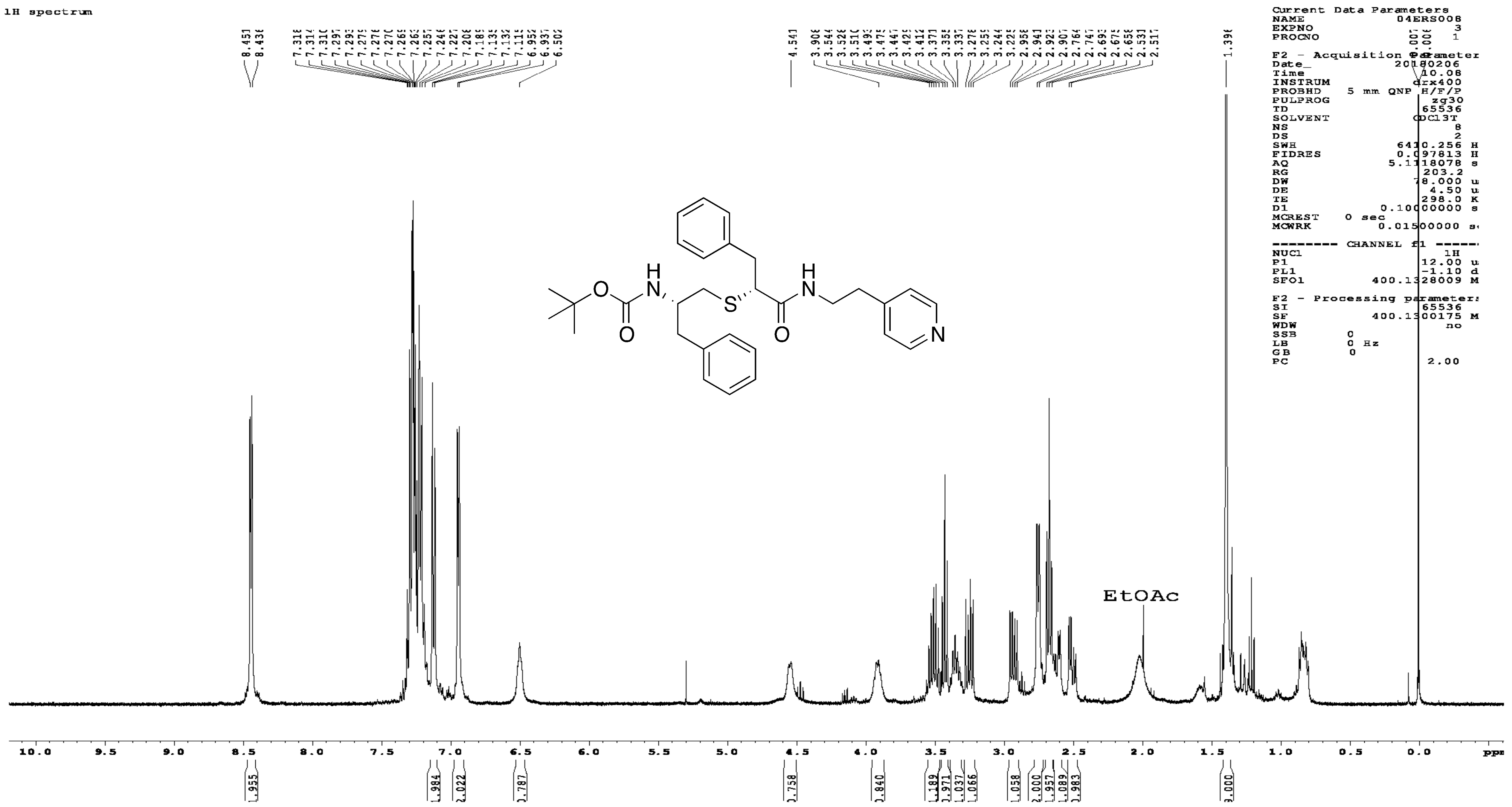
1H spectrum
Current Data Parameters
NAME 04ERS008
EXPNO 3
PROCNO 1
F2 - Acquisition Parameters
Date_ 20180206
Time 10.08
INSTRUM drx400
PROBHD 5 mm QNP H/F/P
PULPROG zg30
TD 65536
SOLVENT CDCl3
NS 8
DS 2
SWH 6410.256 Hz
FIDRES 0.097813 Hz
AQ 5.1118078 sec
RG 203.2
DW 78.000 usec
DE 4.50 usec
TE 298.0 K
D1 0.10000000 sec
MCREST 0 sec
MCWRK 0.01500000 sec
CHANNEL f1
NUC1 1H
P1 12.00 usec
PL1 -1.10 dB
SFO1 400.1328009 MHz
F2 - Processing parameters
SI 65536
SF 400.1300175 MHz
WDW no
SSB 0
LB 0 Hz
GB 0
PC 2.00
EtOAc
ppm

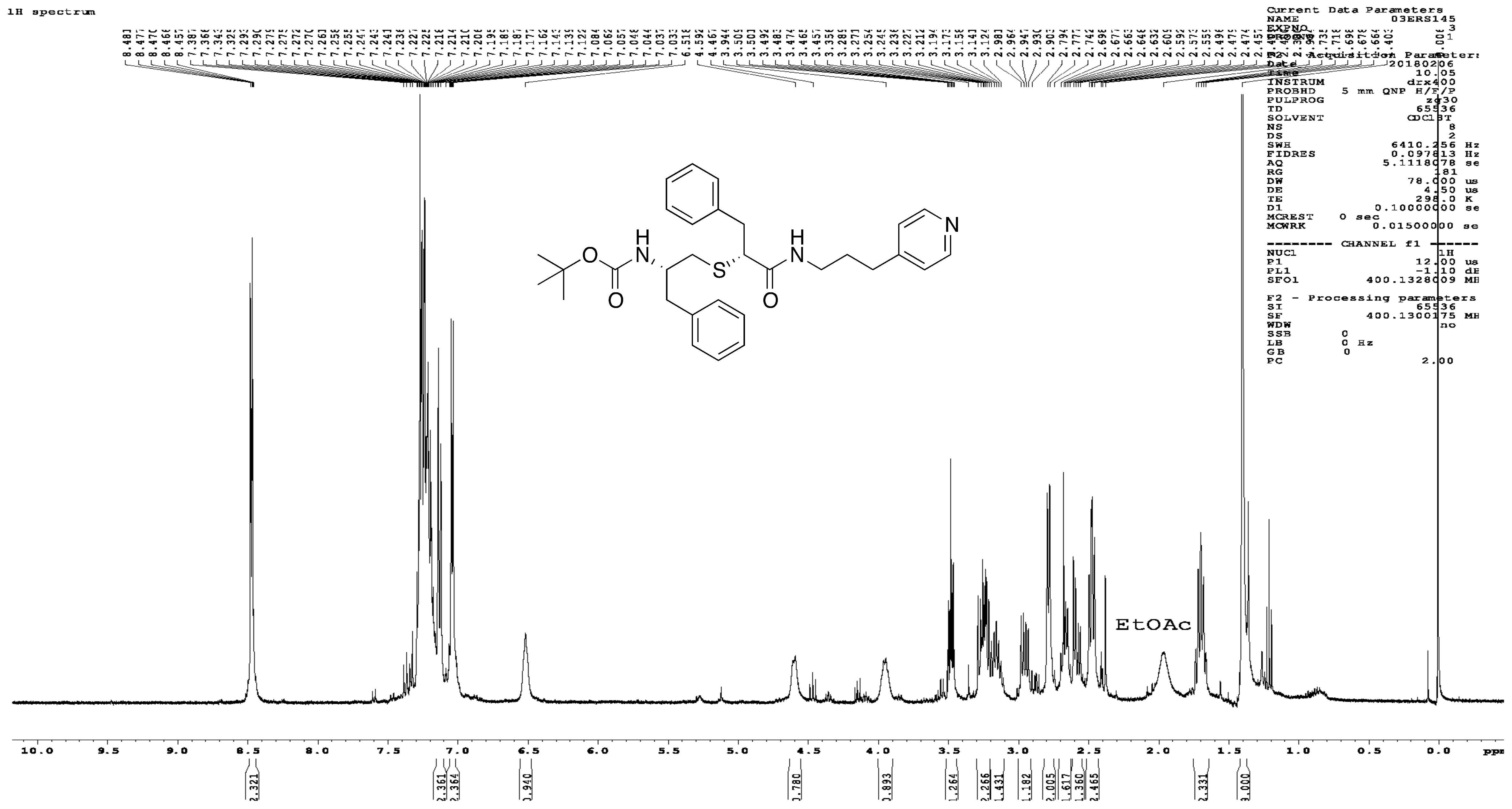

1H spectrum
EtOAc
Current Data Parameters
NAME 03ERS145
EXPNO 3
PROCNO 1
F2 - Acquisition Parameters
Date_ 20180206
Time 10.05
INSTRUM drx400
PROBHD 5 mm QNP H/F/P
PULPROG zg30
TD 65536
SOLVENT CDCl3
NS 8
DS 2
SWH 6410.256 Hz
FIDRES 0.097813 Hz
AQ 5.1118078 sec
RG 181
DW 78.000 usec
DE 4.50 usec
TE 298.0 K
D1 0.10000000 sec
MCREST 0 sec
MCWRK 0.01500000 sec
======== CHANNEL f1 ========
NUC1 1H
P1 12.00 usec
PL1 -1.10 dB
SFO1 400.1328009 MHz
F2 - Processing parameters
SI 65536
SF 400.1300175 MHz
WDW no
SSB 0
LB 0 Hz
GB 0
PC 2.00
ppm

1H spectrum

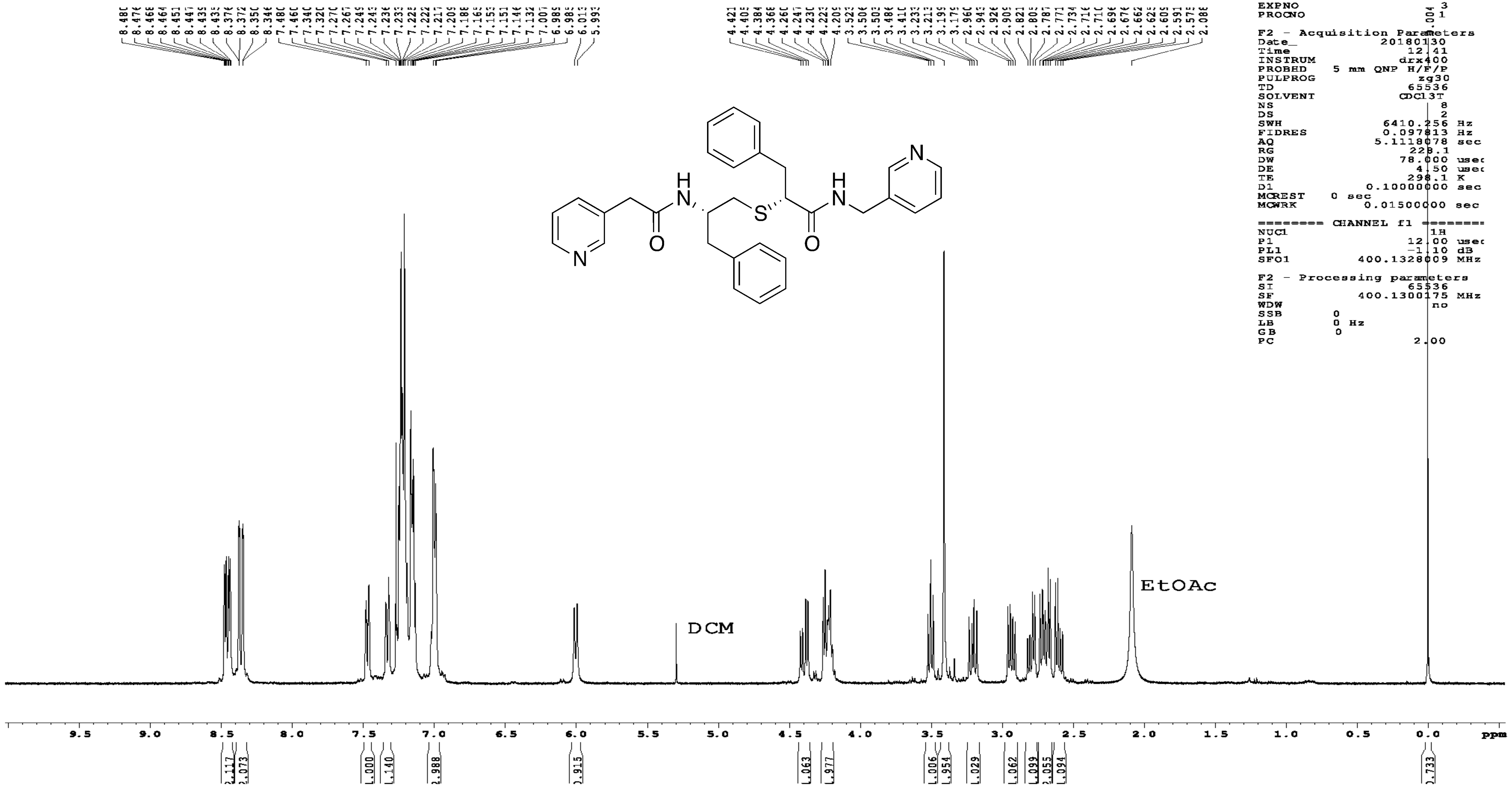

DCM
EtOAc
ppm
Current Data Parameters
NAME 03ERS065
EXPNO 3
PROCNO 1
F2 - Acquisition Parameters
Date_ 20180130
Time 12.41
INSTRUM drx400
PROBHD 5 mm QNP H/F/P
PULPROG zg30
TD 65536
SOLVENT CDCl3T
NS 8
DS 2
SWH 6410.256 Hz
FIDRES 0.097813 Hz
AQ 5.1118078 sec
RG 228.1
DW 78.000 usec
DE 4.50 usec
TE 298.1 K
D1 0.10000000 sec
MCREST 0 sec
MCWRK 0.01500000 sec
======== CHANNEL f1 ========
NUC1 1H
P1 12.00 usec
PL1 -1.10 dB
SFO1 400.1328009 MHz
F2 - Processing parameters
SI 65536
SF 400.1300175 MHz
WDW no
SSB 0
LB 0 Hz
GB 0
PC 2.00

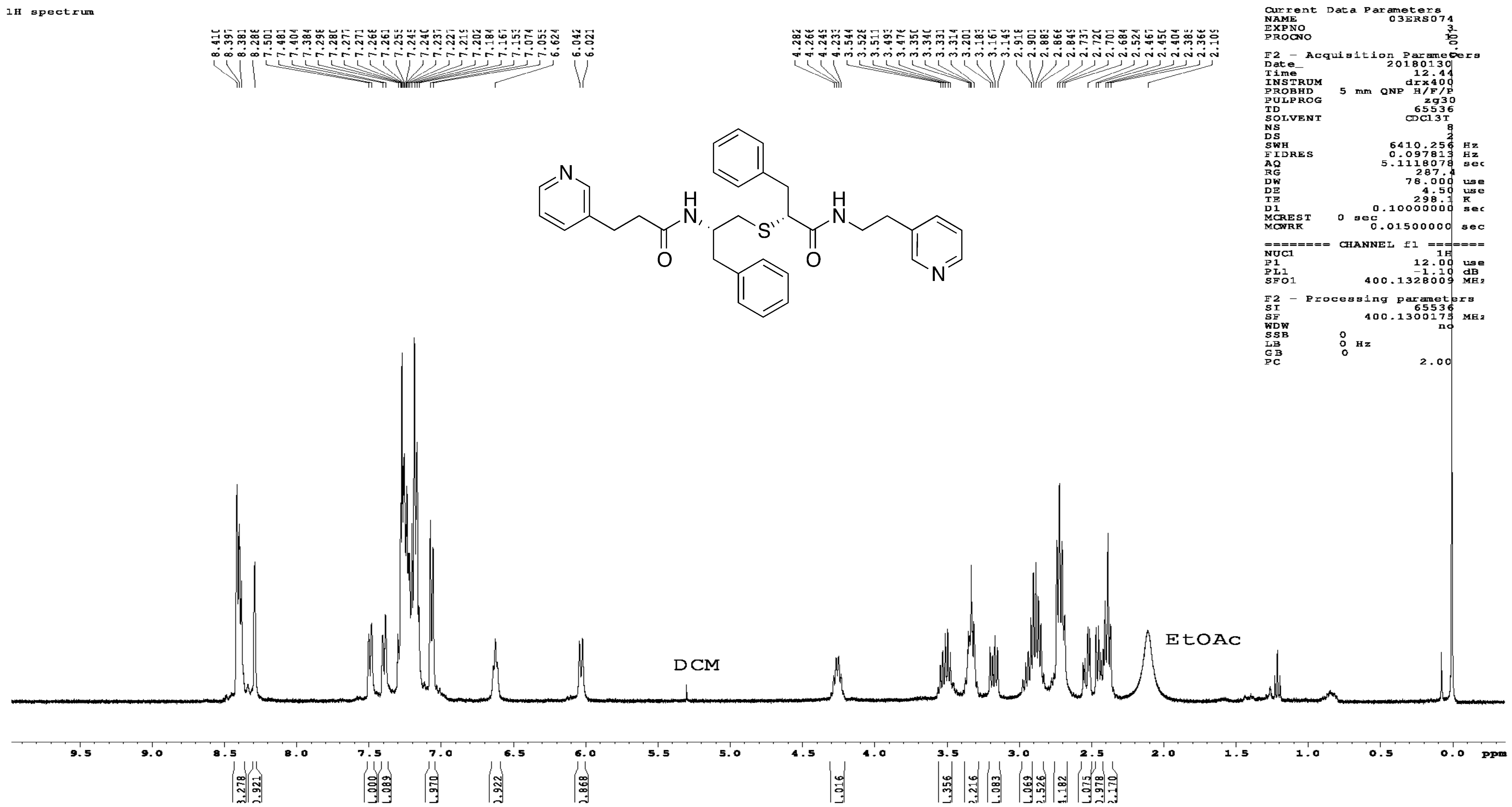
1H spectrum
DCM
EtOAc
ppm
Current Data Parameters
NAME 03ERS074
EXPNO 3
PROCNO 1
F2 - Acquisition Parameters
Date_ 20180130
Time 12.44
INSTRUM drx400
PROBHD 5 mm QNP H/F/P
PULPROG zg30
TD 65536
SOLVENT CDCl3T
NS 8
DS 2
SWH 6410.256 Hz
FIDRES 0.097813 Hz
AQ 5.1118078 sec
RG 287.4
DW 78.000 usec
DE 4.50 usec
TE 298.1 K
D1 0.10000000 sec
MCREST 0 sec
MCWRK 0.01500000 sec
======== CHANNEL f1 ========
NUC1 1H
P1 12.00 usec
PL1 -1.10 dB
SFO1 400.1328009 MHz
F2 - Processing parameters
SI 65536
SF 400.1300175 MHz
WDW no
SSB 0
LB 0 Hz
GB 0
PC 2.00

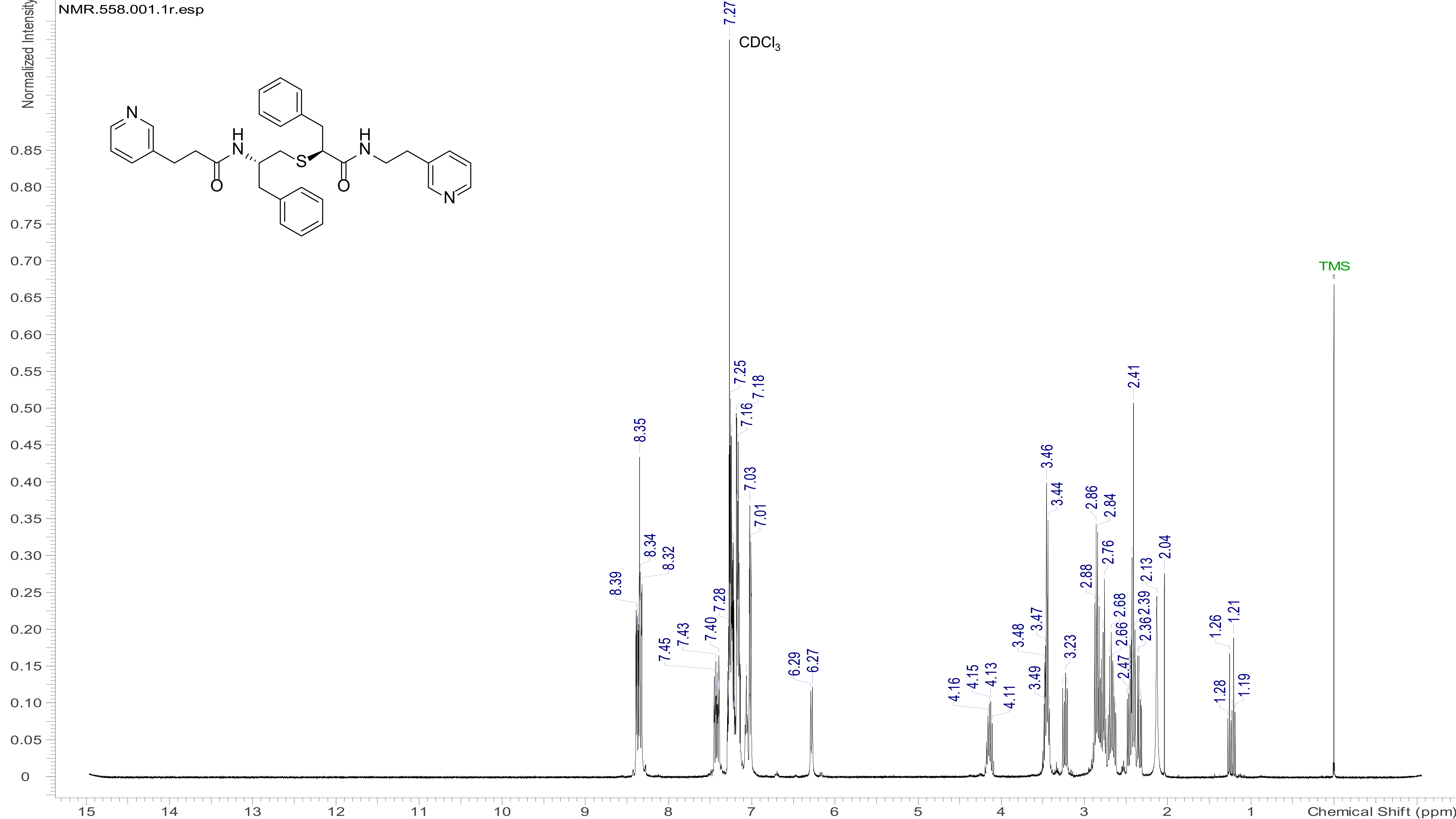
NMR.558.001.1r.esp
Normalized Intensity
Chemical Shift (ppm)
CDCl3
TMS
8.39
8.35
8.34
8.32
7.45
7.43
7.40
7.28
7.27
7.25
7.18
7.16
7.03
7.01
6.29
6.27
4.16
4.15
4.13
4.11
3.49
3.48
3.47
3.46
3.44
3.23
2.88
2.86
2.84
2.76
2.68
2.66
2.47
2.41
2.39
2.36
2.13
2.04
1.28
1.26
1.21
1.19

1H spectrum

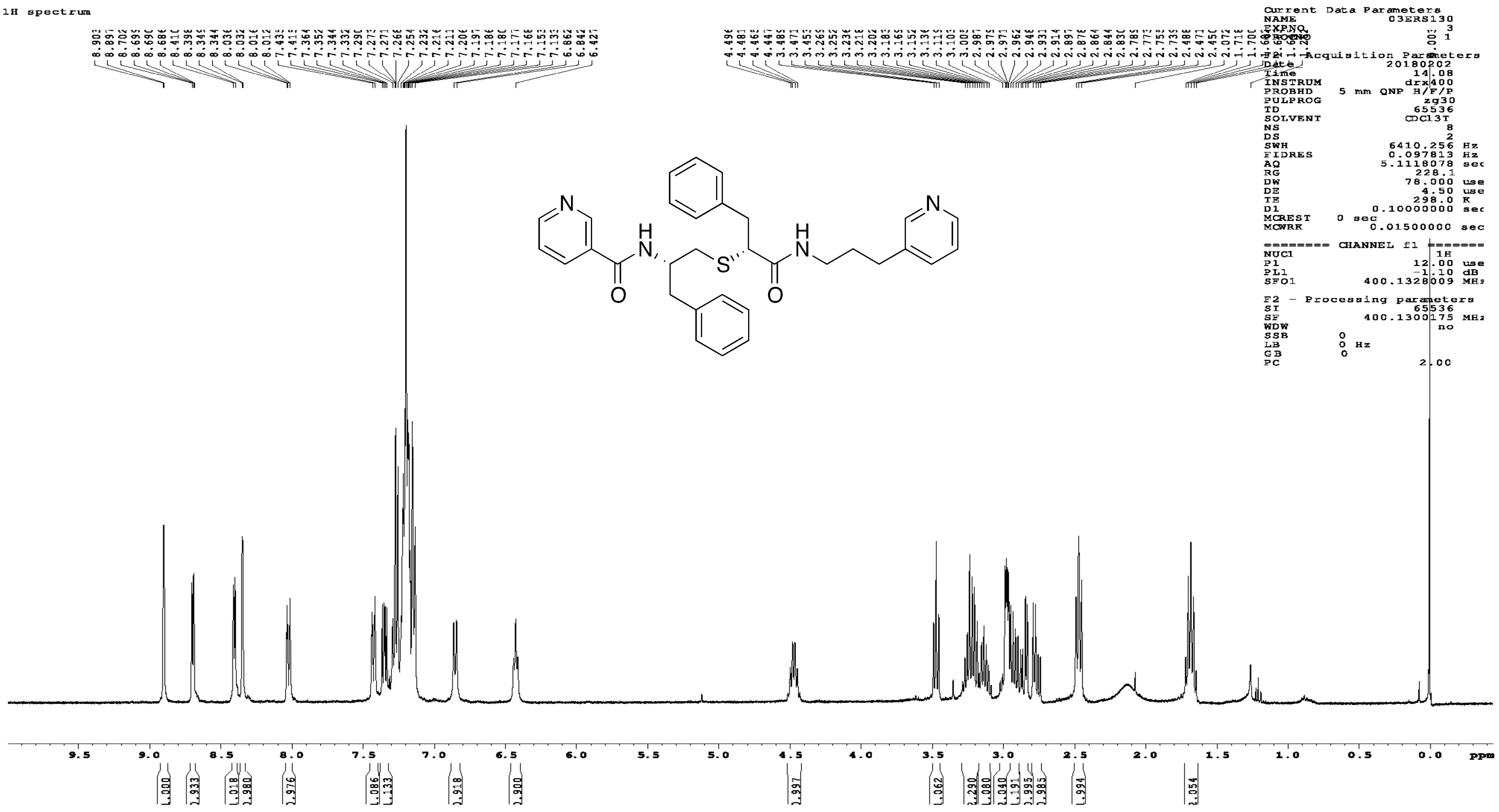
Current Data Parameters
NAME 03ERS130
EXPNO 3
PROCNO 1
F2 - Acquisition Parameters
Date_ 20180202
Time 14.08
INSTRUM drx400
PROBHD 5 mm QNP H/F/P
PULPROG zg30
TD 65536
SOLVENT CDCl3T
NS 8
DS 2
SWH 6410.256 Hz
FIDRES 0.097813 Hz
AQ 5.1118078 sec
RG 228.1
DW 78.000 use
DE 4.50 use
TE 298.0 K
D1 0.10000000 sec
MCREST 0 sec
MCWRK 0.01500000 sec
======== CHANNEL f1 ========
NUC1 1H
P1 12.00 use
PL1 -1.10 dB
SFO1 400.1328009 MHz
F2 - Processing parameters
SI 65536
SF 400.1300175 MHz
WDW no
SSB 0
LB 0 Hz
GB 0
PC 2.00
9.5 9.0 8.5 8.0 7.5 7.0 6.5 6.0 5.5 5.0 4.5 4.0 3.5 3.0 2.5 2.0 1.5 1.0 0.5 0.0 ppm

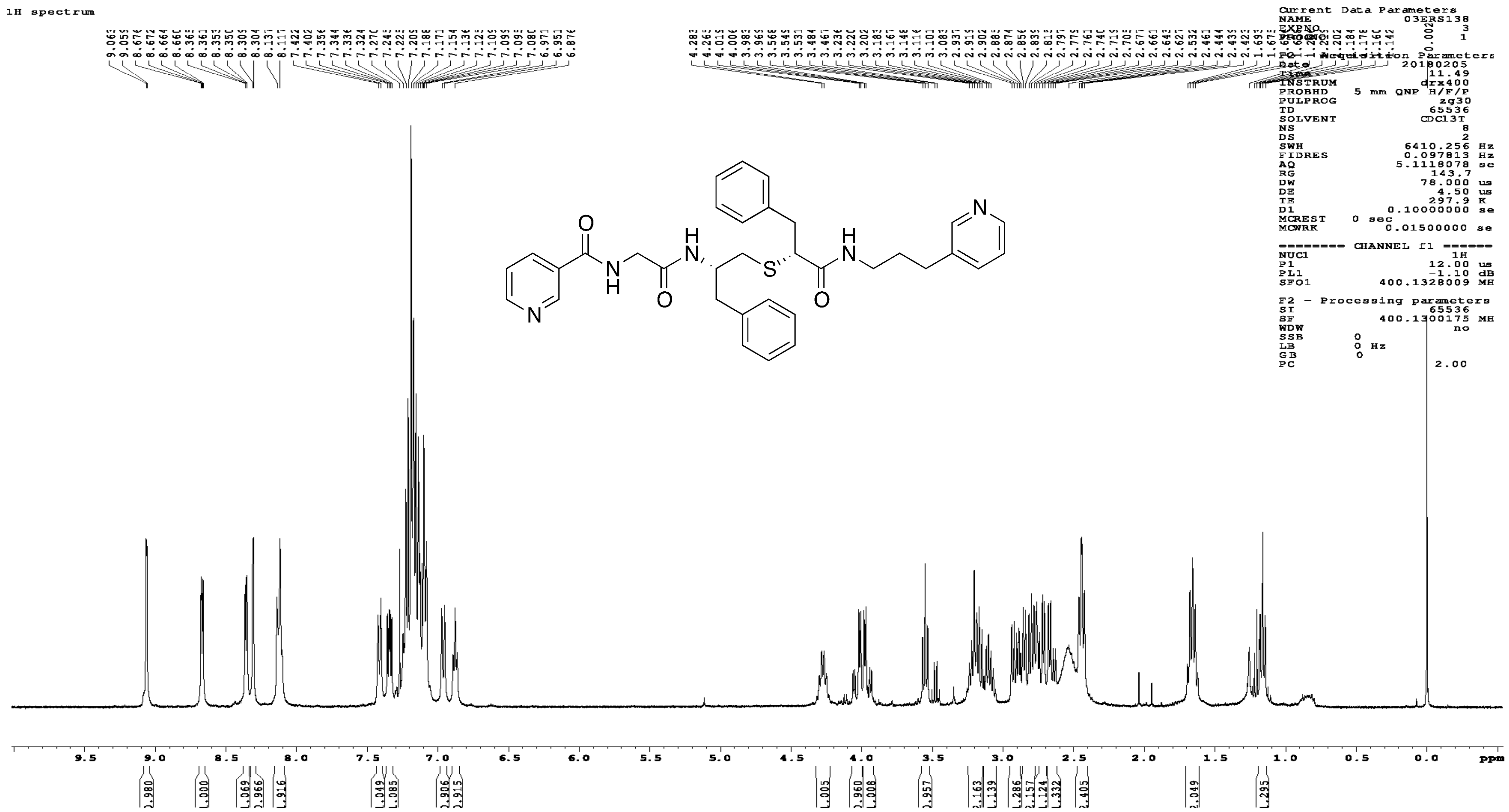
1H spectrum
Current Data Parameters
NAME 03ERS138
EXPNO 3
PROCNO 1
F2 - Acquisition Parameters
Date_ 20180205
Time 11.49
INSTRUM drx400
PROBHD 5 mm QNP H/F/P
PULPROG zg30
TD 65536
SOLVENT CDCl3T
NS 8
DS 2
SWH 6410.256 Hz
FIDRES 0.097813 Hz
AQ 5.1118078 se
RG 143.7
DW 78.000 us
DE 4.50 us
TE 297.9 K
D1 0.10000000 se
MCREST 0 sec
MCWRK 0.01500000 se
======== CHANNEL f1 ======
NUC1 1H
P1 12.00 us
PL1 -1.10 dB
SFO1 400.1328009 MH
F2 - Processing parameters
SI 65536
SF 400.1300175 MH
WDW no
SSB 0
LB 0 Hz
GB 0
PC 2.00
ppm
